# Applying Convergent Immunity to Innovative Vaccines Targeting *Staphylococcus aureus*

**DOI:** 10.3389/fimmu.2014.00463

**Published:** 2014-09-26

**Authors:** Michael R. Yeaman, Scott G. Filler, Clint S. Schmidt, Ashraf S. Ibrahim, John E. Edwards, John P. Hennessey

**Affiliations:** ^1^Department of Medicine, David Geffen School of Medicine at UCLA, Los Angeles, CA, USA; ^2^Division of Infectious Diseases, Harbor-UCLA Medical Center, Torrance, CA, USA; ^3^Division of Molecular Medicine, Harbor-UCLA Medical Center, Torrance, CA, USA; ^4^St. John’s Cardiovascular Research Center, Los Angeles Biomedical Research Institute at Harbor-UCLA Medical Center, Torrance, CA, USA; ^5^NovaDigm Therapeutics, Inc., Grand Forks, ND, USA

**Keywords:** *Staphylococcus aureus*, vaccines, NDV-3, Als3, convergent immunity, convergent antigen

## Abstract

Recent perspectives forecast a new paradigm for future “third generation” vaccines based on commonalities found in diverse pathogens or convergent immune defenses to such pathogens. For *Staphylococcus aureus*, recurring infections and a limited success of vaccines containing *S. aureus* antigens imply that native antigens induce immune responses insufficient for optimal efficacy. These perspectives exemplify the need to apply novel vaccine strategies to high-priority pathogens. One such approach can be termed *convergent immunity*, where antigens from non-target organisms that contain epitope homologs found in the target organism are applied in vaccines. This approach aims to evoke atypical immune defenses via synergistic processes that (1) afford protective efficacy; (2) target an epitope from one organism that contributes to protective immunity against another; (3) cross-protect against multiple pathogens occupying a common anatomic or immunological niche; and/or (4) overcome immune subversion or avoidance strategies of target pathogens. Thus, convergent immunity has a potential to promote protective efficacy not usually elicited by native antigens from a target pathogen. Variations of this concept have been mainstays in the history of viral and bacterial vaccine development. A more far-reaching example is the pre-clinical evidence that specific fungal antigens can induce cross-kingdom protection against bacterial pathogens. This trans-kingdom protection has been demonstrated in pre-clinical studies of the recombinant *Candida albicans* agglutinin-like sequence 3 protein (rAls3) where it was shown that a vaccine containing rAls3 provides homologous protection against *C. albicans*, heterologous protection against several other *Candida* species, and convergent protection against several strains of *S. aureus*. Convergent immunity reflects an intriguing new approach to designing and developing vaccine antigens and is considered here in the context of vaccines to target *S. aureus*.

## Introduction

*Staphylococcus aureus* infections are ubiquitous, potentially life-threatening, and a rising public health issue (1–3). On an individual basis, skin and skin structure infections (SSSI) due to *S. aureus* are among the most frequent of clinical complaints and often complicate unrelated medical procedures (4, 5). Based on 2005 data, over 14 million individuals sought clinical care for SSSI that year in the United States alone (6). More recent data estimate the actual incidence of *S. aureus* infections to be 600 per 100,000, projecting to exceed 1.5 million SSSI due to *S. aureus* each year in the United States (7). Of these, approximately 80,000 yield life-threatening invasive infections annually in the U.S (8–10). Moreover, nearly 15% of patients (roughly 12,000 per year) contracting invasive *S. aureus* succumb to this infection (11). From a broader perspective, the widespread use of antibiotics to treat SSSI is costly and raises the selection pressure favoring increasing drug resistance (12). Methicillin-resistant *S. aureus* (MRSA) strains are now common agents of community-based outbreaks (1, 3, 13). Thus, despite a diminishment in MRSA infections in adults in recent years (9), the incidence of invasive infections due to MRSA remains unacceptably high. In contrast to adults, no significant reduction in healthcare-associated MRSA infections has been observed in children (14). To the contrary, populations vulnerable to *S. aureus* infections extend beyond the immune compromised, and increasingly include otherwise healthy populations for which no endogenous risk factors have been identified (15, 16).

Beyond SSSI, invasive infections due to *S. aureus* are life-threatening and increasingly impervious to even the most modern antibiotics. Infections of skin and skin structure, along with mucocutaneous colonization burden also impose significantly greater risk of invasive infections. Compared to non-carriers, elderly men with high-burden of MRSA nasal colonization develop infections at a fourfold greater frequency than non-colonized individuals (17). In addition, greater burden of skin and mucosal colonization imparts a greater risk for long-term readmission and mortality in MRSA-colonized veterans (18). Moreover, a history of MRSA-positive clinical culture is a significant positive predictor of risk for community-onset invasive MRSA infection following hospital discharge (19). Further, high density nasal colonization by MRSA also increases the risk of invasive disease (20). The incidence of invasive community-acquired MRSA infections in children increased significantly from 2005 to 2010 (14).

From these perspectives, vaccine-mediated protection against *S. aureus* disease overall, and MRSA infections in particular, holds promise to address significant unmet patient needs, leading to significant public health benefit. Beyond mitigating SSSI, vaccines that reduce nasal or mucocutaneous burden of MRSA are also likely to reduce the risk of life-threatening invasive infections. In addition, use of effective vaccines has the potential to enhance antibiotic efficacy or mitigate resistance, by reducing overall use and allowing more selective application of these drugs. Thus, efficacious vaccines targeting *S. aureus* are urgently needed.

## Insights from Natural Host Defense Against *S. aureus*

A primary factor likely resulting in limited success to date in development of an efficacious vaccine against *S. aureus* is an incomplete understanding of key host-defense mechanisms responsible for natural protective immunity. Immunologic determinants relevant to host defense against *S. aureus* infection may be organized into recognition, regulation, or effector systems. Optimization of these systems, individually and synergistically, is necessary for efficacy in novel vaccines targeting this organism.

### Mediation of immune recognition

Pattern recognition receptors [PRRs; e.g., toll-like receptors (TLRs) or nucleotide-binding oligomerization domain like receptors (NLRs)] and their ligation by cognate pathogen-associated molecular patterns (PAMPs) trigger specific signal transduction pathways. These circuitries include myeloid differentiation factor-88 (MyD88), IL-1 receptor-associated kinase (IRAK), inhibitor of κB kinase (IκBK), and nuclear factor κB (NFκB) activation cascades. Their activation yields up-regulation of host-defense peptide and cytokine expression (21–24). Deficient TLR-mediated responses (25) emphasize the importance of these circuits in rapid defense against *S. aureus* infection. Immune dysfunctions that render patients at increased risk of *S. aureus* infection (26–28) include deficient TLR or TLR-mediated response pathways [e.g., MyD88, IRAK-4, IL-1R (21, 25, 27)], and dysfunctions in IL-1β induction (29, 30). Insightful reviews of these topics can be found elsewhere (31–33).

### Immune regulation

In 2008, Renner et al. (27) identified a dominant polymorphism in the gene encoding signal transduction/activator of transcription-3 (STAT3) that was associated with recurring infections due to *S. aureus* or other opportunistic pathogens. Because STAT3 is key to the differentiation of Th17-polarized T cells, this condition is associated with recurring infections often observed in Job’s Syndrome (also called Hyper-IgE syndrome or Buckley Syndrome). In response to IL-6 and TGFβ or IL-23 elaborated by antigen-presenting cells (APC), Janus-activated kinase-2 (JAK2) phosphorylates and activates STAT3. In turn, the phosphorylated STAT3 (STAT3P) activates the transcription factor RAR-related orphan receptor γτ (RORγτ), and to a lesser extent RORγα. These events lead to expression of IL-17A and other members of the Th17 cytokine circuitry, including IL-22. We and other groups have demonstrated that the IL-17 pathway (IL-17A, IL-22, host-defense peptides) is of particular relevance to immune defense against *S. aureus* infection. Interestingly, αβ and γδ CD4+ T cells may contribute differently to induce the Th17 pathway in distinct tissue contexts (34, 35). Excellent reviews of this concept can be found elsewhere (31, 36) As discussed below, it is important to note that both IL-17 and IL-22 significantly influence the function of innate immune effector systems against *S. aureus*, including neutrophils and host-defense peptides.

Resident γδ T cells are key to early containment and clearance of *S. aureus* at the interface of skin and mucosa (37). However, γδ T cells do not appear to induce IL-22 in relation to the IL-17 pathway in mouse models (38), and mice deficient in αβ T cells have dysfunctional abscess formation in *S. aureus* wound infection models (39). Moreover, IL-17A/F deficiency predisposes to spontaneous dermatologic infection, but not hematogenous infection by *S. aureus* in some mouse models (40). Thus, vaccines that induce protective CD4+ αβ and γδ T cell-mediated responses may optimize protective efficacy against *S. aureus* SSSI.

In addition to the Th17 response, a Th1 response may also play a role in defense against infection due to *S. aureus*. Our laboratory and others have shown that interferon-γ (IFN-γ) is actively induced in response to *S. aureus* infection (41). Further, Cho et al. showed that IL-1β is of special importance to effective host defense of the skin (42). Paralleling the activation of STAT3 in Th17 responses, Th1 responses are mediated by STAT4 activation in response to signaling by IFN-γ and IL-12. Phosphorylation of STAT4, largely via JAK1, induces expression of T-box protein expressed in T cells (Tbet), which then promotes interferon regulatory factor-9 (IRF9) to up-regulate type I (IFN-α and β) and type II (IFN-γ) interferons. Although the role of type I interferons in host defense against *S. aureus* is not well understood, IFN-γ is likely to play a role in cell-mediated immunity against this and other opportunistic pathogens. It is notable that infections due to *S. aureus* are significantly more common in individuals affected by HIV than in HIV-negative populations. This relationship supports the importance of cell-mediated adaptive immunity and T-cell regulation in effective host defense against this pathogen. Interestingly, in some models, neutralization of IFN-γ may actually enhance the host defense against recurrent *S. aureus* skin infection (43), potentially by de-repression of Th17 pathways.

It should also be emphasized that natural infection caused by *S. aureus* elicits a robust antibody response in immune competent individuals. In contrast to the Th17 or Th1 paradigms, a Th2 response plays a key role in the induction of antibodies in response to *S. aureus*. In this circuit, IL-4, IL-5, and IL-13 are the signature cytokines for CD4+ T-cell polarization to a Th2 response. The signaling pathway that mediates this response involves STAT6 activation of the GATA3 transcription factor (44). Importantly, colonization in the absence of frank infection may be insufficient to induce high Ab levels. Alternatively, even if baseline Ab levels are elevated in healthy individuals as a result of colonization, infection due to *S. aureus* induces a further rapid increase in Ab levels. Thus, the exposure and response of B cells to staphylococcal antigens is an acute aspect of the adaptive immune response to this organism. As will be discussed below, whether a protective or durable anamnestic response occurs in response to *S. aureus* infection remains a central question in natural infection, and is a key goal of novel *S. aureus* vaccines.

### Immune effectors

In healthy individuals, *S. aureus* colonizes human skin and mucosa without causing overt disease, and without inflammatory host defenses. This fact suggests differences in fundamental aspects of host defense against *S. aureus* colonization versus invasion. Moreover, specific molecular or cellular effectors, and their coordination, likely differ in distinct tissues (e.g., mucocutaneous versus hematogenous), or in different stages of infection (acute versus chronic). Mindful of these caveats, effectors involved in anti-staphylococcal host defense of relevance to potential mechanisms of protective vaccines are reviewed below.

#### Mechanochemical barrier

Initial insights from the Human Microbiome Project (NIH) have affirmed that *S. aureus* is a predominant and common member of the human microbiome (45). The structure and chemical composition of intact skin are key features that normally ward off *S. aureus* opportunistic infection. From the continuous exfoliation of the epidermis, to its antimicrobial chemistry as reflected in sebum, salt, and pH conditions shaped by sebaceous, eccrine, and apocrine glands, the human skin imposes a mechanochemical barrier to *S. aureus* invasion (46). Additionally, ceramides, squalene, and wax esters likely contribute to cutaneous defense against *S. aureus* (47). Such factors have not been traditionally considered accessible to vaccines or anti-infective immunotherapies.

#### Host-defense peptides

Human skin and mucosa are perfused with a variety of host-defense peptides. Principal structural classes are the defensins, cathelicidins, and other molecules including S100A7 (psoriacin) and dermcidin (48). Prototypic α-defensin, β-defensin, and cathelicidin class molecules are human neutrophil peptide-1 (hNP-1), β-defensin-2 (hβD-2), and LL-37, respectively. Microbicidal chemokines (*kinocidins*) derived from platelets and other cells also defend against *S. aureus* and other infections (24, 49). The regulation, structural biology, and mechanisms of action of these and related molecules are the topics of other reviews (50–52). New evidence is emerging that host-defense peptides may be inducible by vaccination in protection against *S. aureus* SSSI (see below).

#### Humoral immunity

B lymphocytes and antibodies may contribute to protection against mucocutaneous colonization or invasive infection caused by *S. aureus*. First, functional antibodies can promote opsonophagocytosis (FcγRII equivalent receptor-mediated), neutralize extracellular virulence determinants (e.g., secreted exotoxins), or potentiate targeted complement fixation (e.g., IgG dimers or IgM via the classical pathway). For example, the anti-Panton–Valentine leukocidin antibody (anti-LukAB) emerges in transition from acute-phase to convalescent sera in children with invasive *S. aureus* infection (53). Second, B cells are now increasingly recognized as integral to antigen presentation (54, 55) and cytokine conditioning of T-cell responses (56). It should also be recognized that generation of IgG antibody subclasses 1 or 3 (IgG_1_ or IgG_3_) in humans is dependent upon CD4+ T-cell activation of B-cell subclass switching. Thus, such antibody responses are subordinate to T-cell regulation, emphasizing the essential roles of T cells in humoral and cell-mediated immunity to *S. aureus*.

The potential risks of inducing opsonophagocytic antibodies in patients having overt or cryptic granulocyte dysfunctions should also be recognized. *S. aureus* is a facultative intracellular pathogen capable of at least temporary survival in the harsh phagolysosome of the neutrophil. Thus, even if antibody targeting *S. aureus* is capable of promoting opsonophagocytosis *in vitro*, unless the granulocyte can rapidly kill the organism, opsonization could simply facilitate phagocytosis for immune avoidance and cryptic dissemination. This mechanism of immune subversion by *S. aureus* has been hypothesized in P47phox null mice by Pancari et al. (57).

#### Complement

Complement cascades afford molecular bridges between innate and adaptive immunity. Lectin and alternative-pathway complement fixation can both occur in response to PAMPs such as teichoic acid and peptidoglycan on the *S. aureus* surface (58). By comparison, classical complement fixation leverages the humoral response to guide complement targeting of *S. aureus*. Here, the C1qrs complex targets antigen-specific antibody on the organism surface. Complement fixation also yields opsonins such as C3b and C5b, which decorate the organism for opsonophagocytosis by professional leukocytes. Furthermore, anaphylatoxin products of complement protein cleavage (e.g., C3a and C5a) support leukocyte chemotaxis to sites of *S. aureus* infection. Activation of the antimicrobial mechanisms of these cells (reactive oxygen species, hypohalous reactants, cleavage of antimicrobial peptides, granule mobilization, etc.) occurs *en route* to sites of infection (59).

#### Neutrophils and other host cells

Neutrophils (or polymorphonuclear leukocytes) represent the predominant cellular effectors of host-defense against *S. aureus*. This fact is supported by numerous clinical conditions in which lack of functional neutrophils, or dysfunctions in neutrophils are associated with increased risks of *S. aureus* infection: (a) neutropenia (60–63); (b) dysfunctions in granulocyte oxidative burst [e.g., chronic granulomatous disease; (64, 65)]; (c) dysfunctions in Th17 polarization, which recruits/activates neutrophils [e.g., Job Syndrome; (66, 67)]; and (d) dysfunctions in leukocyte adhesion molecule expression or function as required for neutrophil margination and diapedesis to target sites of infection [e.g., LAD-1 and -2; (68, 69)]. Less clear are the roles of cells in protection against *S. aureus* infection. These include the monocyte/macrophage lineage, natural killer (NK), and perhaps even CD8^+^ T cells. For example, up to 50% of *S. aureus* organisms that are phagocytized by neutrophils survive intracellularly. Normally, macrophages in-turn efferocytose *S. aureus*-containing neutrophils, thereby enabling effective clearance. However, intra-neutrophil *S. aureus* appear to cause neutrophil up-regulation of CD47, preventing their effective efferocytosis by macrophages, thus facilitating survival and potential metastatic seeding of *S. aureus* (70). Efficacious vaccines will need to overcome such *S. aureus* survival mechanisms.

#### Other effectors

The direct antimicrobial activities of kinocidins (microbicidal chemokines) are likely to play important roles in defense against *S. aureus* infection (49). For example, through IL-12 elaboration, dendritic cells appear to induce and coordinate CXC, CC, and other kinocidins (71). Recent evidence has also uncovered previously unforeseen relationships that offer insights into host-defense against *S. aureus* on one hand, and polymicrobial host–pathogen relationships on another. For example, infection with influenza suppresses NADPH oxidase-dependent opsonophagocytic clearance of bacteria by macrophages and neutrophils in mouse models (72). In turn, susceptibility to secondary MRSA infection is significantly increased. On another front, invariant natural killer/T-cell (iNKT) receptor-expressing cells can produce IL-17A independently of IL-6 co-stimulation. Hence, iNKT cells also may promote protective inflammatory responses to *S. aureus* infection. Further, IL-1 and IL-23 elaboration is necessary for sustained IL-17A/F and IL-22 generation by iNKT cells in response to *S. aureus* (73). Moreover, IL-1 and IL-23 generated by PAMP-stimulated dendritic cells can also induce *in vitro* IL-1 and IL-23 secretion from NK1.1^−^ cells, which are iNKT cells principally found in the skin and peripheral cutaneous lymph nodes. In parallel, CD8+ T cell-mediated clearance of *S. aureus*-infected host cells has been reported in a murine model (74). Mice exposed to heat-killed *S. aureus* generate robust CD8+ T-cell responses when CD40 ligand is co-stimulated with specific antibody, yielding protective efficacy in a mastitis model. Such examples suggest that historically unappreciated molecules and cells are likely integral to host defense against *S. aureus*, and may be integral to creating efficacious vaccines.

## The Challenge of Normal Flora in Vaccine Development

Co-evolution with mammals has uniquely enabled *S. aureus* to occupy a specialized niche as a part of the normal bacterial microbiota (75). Thus, the human immune system is faced with a balancing act: hold the organism at bay, without evoking unnecessary inflammation. Yet, why *S. aureus* transitions from a harmless commensal to an opportunistic pathogen remains a mystery in many cases? For example, acquired deficits in host-defense mechanisms (e.g., neutropenia) are clearly associated with increased risk of invasive *S. aureus* infection. However, more subtle or even cryptic conditions, alone or in combination, may also render a host at increased risk of infection. Further, certain virulence factors of *S. aureus* may correlate with an increased propensity to cause an opportunistic infection, as might interactions with other normal flora. Some of the factors that could mitigate such an opportunistic infection are discussed below.

### Influence of other vaccines on *S. aureus* colonization

Vaccines targeted to pathogens other than *S. aureus* have been observed to produce significant increases in colonization by *S. aureus* in vaccine recipients. Specifically, use of the 7-valent pneumococcal conjugate vaccine, which reduces both nasopharyngeal colonization as well as disease due to serotypes included in the vaccine, can increase the frequency of *S. aureus* colonization in those receiving the vaccine versus unvaccinated controls (10.1 versus 5.0%, respectively) (76). Additionally, the use of live attenuated influenza vaccine has been shown to increase the carriage density of and duration of colonization by *S. aureus*, much like what has been observed with wild-type influenza virus (77). This phenomenon is most likely due to a reestablishment of the equilibrium of the microbiome, but could also be due to a deviation of the immunological profile of some individuals such that they become better hosts for *S. aureus*.

### Impact of microbiome on immune polarization

The impact of the human microbiome on the immunological posture of individuals and their response to vaccines is a relatively new field. Explorations such as those by Eloe-Fadrosh et al. (78) provide insight that the microbiome can greatly influence polarization of the immune system. In their particular study, individuals harboring a more diverse and complex microbiome community exhibited a multiphasic cell-mediated immune response to an oral typhoid vaccine. In contrast, Ferreira et al. (79) speculated that the more diverse microbiome of individuals from developing countries may account for the suppressed immune response to several vaccines as is observed relative to more developed countries.

Observations that commensal bacterial and commensal pathogens leave an immunological “footprint” in the form of detectable humoral and cell-mediated immune response to some surface proteins are common [e.g., Ref. (80–82)] and not altogether unexpected. Combined, these observations suggest that the mere presence of *S. aureus* in the microbiome should not be expected to be sufficient to explain or predict the readiness of a given immune system to respond to a vaccine targeting *S. aureus*.

### Impact of immunization on microbiome

It is well established that vaccines targeting select serotypes of bacterial pathogens (e.g., *Streptococcus pneumoniae, Neisseria meningitidis*) are very successful at reducing or eliminating serotypes that are represented in the vaccine. However, it is also evident that such vaccination can result in serotype replacement with non-vaccine serotypes of the same or related organisms [e.g., Ref. (83, 84)]. Additionally, potential synergistic relationships between specific commensal pathogens, such as observed between *S. aureus* and *Candida albicans* (85), *C. albicans* and *Pseudomonas aeruginosa* (86), and *P. aeruginosa* and *S. aureus* (87). These relationships suggest that altering the microbiome composition via immunization may have broader impact by creating a less favorable environment for synergistic pathogens.

### Experience in prior use of native *S. aureus* antigens

To date, there have been at least 19 *S. aureus* vaccine clinical studies conducted in the US (see Table 1) that have evaluated over 20 different antigens (see Table 2). Although much of the data remain proprietary, all of these antigens had to have been successfully evaluated in one or more animal challenge studies and shown to be safe and immunogenic when evaluated in pre-clinical toxicity studies to be considered for clinical evaluation. Thus, insufficient immune response in humans may be one factor that has limited the number of these vaccine candidates advancing to Phase 2 studies. To date, no *S. aureus* vaccines have been demonstrated to have efficacy for any specific disease indication in humans. For example, two of the vaccines that were advanced to Phase 2b/3 studies did not demonstrate efficacy (88, 89) and one of those, using the IsdB antigen, revealed potentially significant safety issues in cardiothoracic surgery patients (89). All of these antigens induce antibodies in human vaccines, many of which have been shown to have functional activity against *S. aureus* isolates. Furthermore, several of these vaccines have been demonstrated to induce T-cell responses, including IL-17A and IFN-γ. Even so, there is still no clear evidence for or consensus on surrogate markers of protection against any *S. aureus* indication. Recently, a tetravalent *S. aureus* vaccine was advanced into a Phase 2/3 clinical trial (NCT01643941; see Table 1). Whether this vaccine will be more efficacious than its predecessors remains to be determined.

**Table 1:** **Vaccines containing *S. aureus* antigens that have been evaluated in human clinical trials as reported on clinicaltrials.gov**.

Sponsor	Vaccine	Antigens	Clinicaltrials.gov identifier	Clinical Study Phase
Nabi	StaphVAX^a^	CP8 conjugate, CP5 conjugate	NCT00071214	3
			NCT00130260	3
			NCT00211900	3
			NCT00211913	3
			NCT00211926	3
			NCT00211965	3
			NCT00211991	3
GSK	GSK2392	4-component	NCT01160172	1
Pfizer	SA3Ag	CP8 conjugate, CP5 conjugate, clumping factor A	NCT01018641	1
	SA4Ag	Same as SA3Ag plus manganese transporter C	NCT01364571	1
	SA4Ag and SA3Ag		NCT01643941	1, 2
Merck	V710^a^	Iron surface determinant B	NCT00303069	1
			NCT01324440	1
			NCT00735839	1
			NCT00822757	1
			NCT00572910	2
			NCT00518687	2, 3
NIAID	STEBvax	Enterotoxin B	NCT00974935	1
Uniformed services	Monovalents	Alpha toxin	NCT01011335	1, 2
University of the Health Sciences		Panton–Valentine leukocidin toxoid	
	Bivalent	Alpha toxin, Panton–Valentine leukocidin toxoid	

*^a^Development terminated*.

*CP, capsular polysaccharide*.

**Table 2:** **Vaccine antigens targeting *S. aureus* that have been evaluated in human clinical trials**.

Antigen	Description	Sponsor	Clinical study phase	Reference
CP8 conjugate CP5 conjugate PVL Hla	CP8 conjugated to rEpA CP5 conjugated to rEpA Panton-Valentine Leukocidin toxoid Alpha hemolysin	GSK		No public disclosure. GSK purchased the Nabi pentavalent *S. aureus* vaccine (PentaStaph) in 2011 (http://globenewswire.com/news-release/2011/04/27/445328/219830/en/Nabi-Biopharmaceuticals-Completes-Final-PentaStaph-TM-Milestone.html)
SEB	Enterotoxin B	NIAID		(90, 91)
Protein 1 Protein 2 Protein 3 Protein 4	No public disclosure of the identity of the antigens.	Novartis	1	N/A
Cbp Clf Fbp Eap	Collagen-binding protein Clumping factor Fibronectin binding protein Extracellular adherence protein	Vaccine Research International		http://www.vri.org.uk/PhaseITrial.pdf
IsdB	Iron surface determinant B	Merck^a^		(82, 89)
CP5 conjugate	CP5 conjugated to CRM197	Pfizer	1, 2	(92)
CP8 conjugate	CP8 conjugated to CRM197	
Mntc	Manganese transporter C	
ClfA	Clumping factor A	
rAT rLukS-PV	Alpha hemolysin Panton-Valentine Leukocidin toxoid	Uniformed Services University of the Health Sciences		http://www.empr.com/bivalent-s-aureus-vaccine-demonstrates-good-immunogenicity-and-neutralizing-activity/article/314713/
CP8 conjugate	CP8 conjugated to rEpA	Nabi^a^	1, 2, 3	(93, 94)
CP5 conjugate	CP5 conjugated to rEpA	
CP336 conjugate	CP336 conjugated to rEpA	

*^a^Development terminated*.

*CP, capsular polysaccharide; rEpA, recombinant *P. aeruginosa* exoprotein A; CRM197, cross-reactive material 197; a non-toxic recombinant variant of diphtheria toxin (*Corynebacterium diphtheriae*)*.

## The Paradox of Recurring *S. aureus* Infection

A mystery regarding human infections caused by *S. aureus* is the propensity of this organism to cause recurring infections due to poor efficacy of anamnestic response in a significant proportion of the overall population. There are several potential and non-exclusive explanations for this observation.

### Immune subversion

*Staphylococcus aureus* is known to have a wide array of virulence factors and immune subversion mechanisms. These strategies include dysregulating T-cell responses via superantigens, disabling host-defense peptides via protease elaboration, avoidance of complement and antibody activities by capsular expression, and suppression of phagocyte chemotaxis and phagocytosis. The specific mechanisms of immune subversion by *S. aureus* are the topic of other excellent reviews (95–97). Interestingly, Skurnik et al. recently identified natural antibodies in normal human sera that inhibit antibody targeting the *S. aureus* capsule (98). This observation raises the possibility that anti-idiotypic or other auto-antibodies diminish otherwise protective humoral immunity versus *S. aureus*. Thus, even if a perfectly appropriate and otherwise efficacious natural immune response were to be induced by a vaccine, it may not be fully or even detectably effective due to direct or indirect immune subversion mediated by *S. aureus*.

### Ineffective anamnestic response

An anamnestic response to infection requires the presence and function of long-term immune memory cells. Relevant to *S. aureus* infection, mucosal FCRL4^+^/CD27^−^ B memory cells, as well as lymphoid FCRL4^−^/CD27^+^ B memory cells are likely required for sustained or rapidly inducible antibody formation (99). Beyond B cells, CD45RA^−^/CCR7^+^ memory T cells in lymph nodes, and CD45RA^−^/CCR7^−^effector memory T cells in mucocutaneous settings are believed to contribute to anamnestic responses (100, 101). In recurring infections due to *S. aureus*, it is likely that such anamnestic response mechanisms are either sub-optimal, or subverted by the pathogen. For example, a recent analysis by Fowler and Proctor (102) posits that antibody is not the primary mechanism of host defense against *S. aureus* disease. Their review of the literature yielded no causal relationship connecting IgG deficiency or hypogammaglobulinemia with increased propensity for *S. aureus* infection. Thus, vaccine strategies that solely induce anamnestic humoral responses targeting native antigens face challenges based on natural history of *S. aureus* infection in humans. In parallel, phenol-soluble modulin (PSM) toxins elaborated by *S. aureus* may cause dysfunctional antigen presentation by dendritic cells to memory lymphocytes (103). Moreover, *S. aureus*-induced dysregulation of monocyte responses, along with a decreased central memory CD4+ and CD8+ T-cell response, is associated with invasive infection due to *S. aureus* in pediatric populations (104). From these perspectives, efficacious vaccines targeting *S. aureus* must, by necessity, enhance anamnestic responses in ways that include cell-mediated mechanisms.

### Exploitation of regulatory lymphocytes

In neonates, exposure to *S. aureus* polarizes naive CD4+ T cells to a FOXP3^+^/CD25^+^/CD127^Low^ phenotype, corresponding to a modulated immune response (105). This response likely establishes tolerance to colonization early in life. While such tolerance may prevent a potentially harmful inflammatory response to commensal *S. aureus*, it may also impair effective immune responses to invasion or re-infection. Similarly, a regulatory subset of CD8^+^/CD11c^hi^T cells has been shown to emerge in the setting of *S. aureus* infection (106). This regulatory subset may inhibit appropriate CD4+ T-cell polarization in response to the organism. As above, the capability to overcome exploitation or dysregulation of appropriate immune responses is likely necessary for efficacy in novel vaccines targeting *S. aureus*.

## Applying Convergent Immunity to Innovative *S. aureus* Vaccines

The perspectives above offer broad insight into the multi-factorial and synergistic quality of natural host defense against *S. aureus*. It follows that vaccines affording protective efficacy against this organism will necessarily stimulate optimal immune defenses to target the organism and spare host tissues. To this end, a review of recent studies of vaccine development and evaluation in humans and experimental models is helpful.

### The principle of convergent immunity

Over the span of evolutionary time, humans and microbes have developed highly specialized systems by which they may exploit and protect themselves from one another. A basic premise of protective immunity is that specific antigens in a given pathogen can induce protective immunity to that pathogen, referred to as homologous immunity, and in many cases can afford protection against various strains of the target pathogen, referred to as heterologous immunity [e.g., Ref. (107, 108)]. In a similar context, microbial pathogens have evolved to exploit specific host features for virulence (e.g., adherence and invasion of host cells to initiate the infection process). Thus, diverse pathogens with similar host targets and niches may use similar or identical pathogenesis strategies. The existence of such similar host–pathogen relationships would logically predict overlaps or even convergence in immunological defenses that may be targeted by *convergent vaccine antigens*. These antigens are structural and/or functional homologs from organisms that have convergent structural and/or functional characteristics to those found in the target pathogen. These concepts comprise the principle of *convergent immunity*.

**Figure 1:**
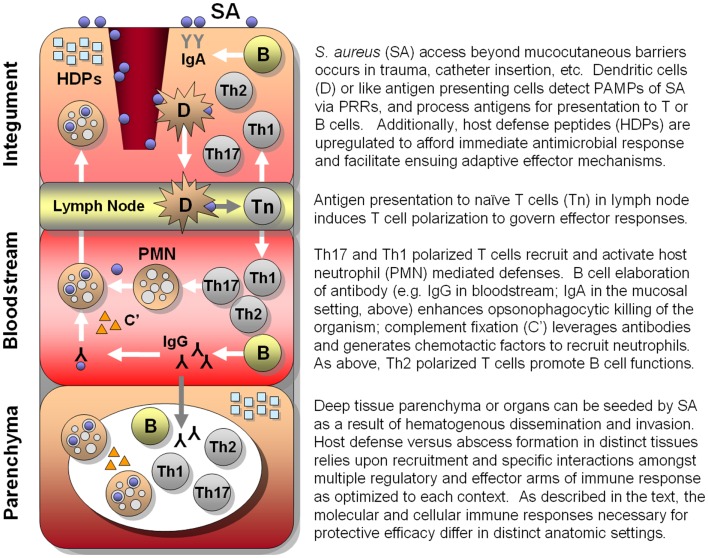
**Hypothesized integrated human immune circuitries in host defense against *S. aureus* infection**. In this illustration, three distinct immunological compartments are shown. Note that lymphocytes, phagocytes, antibody, and molecular effectors (e.g., host-defense peptides, complement, antibody) may be present in each immunological compartment. However, specific profiles and roles of immune effectors may not be identical in each compartment. Vaccines that apply convergent immunity may be designed to optimize those defenses against *S. aureus* that best protect the host in context of specific compartments.

As detailed in the discussion above, the human immune system relies upon an array of PRRs that have been optimized to sense and trigger rapid responses to cognate PAMPs (Figure 1). In turn, immune responses activated by these triggers have evolved to recruit, activate, and regulate immune effectors most capable of neutralizing that particular microbial threat. Even so, co-evolution of normal flora as well as obligate pathogens has allowed microbes the opportunity to evolve specialized strategies by which to avert host defenses, enabling colonization or pathogenesis. Thus, convergent immunogens induce immune processes surmounting those to which microbes have already become adapted or resistant and, in theory, afford protective efficacy.

From this perspective, it follows that the generation of immunogens that stimulate host defenses not typically encountered by a given microbe may have advantages in development of novel vaccines. This concept of convergent immunity is similar to that termed “un-natural” immunity (109, 110). To this end, the era of molecular and cellular technologies has, in effect, accelerated evolution, such that mankind can now rapidly create strategic immunogens that are not known to be present naturally in any microbe. Moreover, novel adjuvants are emerging that provide opportunities to further shape immune responses to convergent antigens. As a result, it may be possible to use convergent immunity strategies to stimulate host defenses that would not be induced otherwise, and which are highly protective against *S. aureus* and other high-priority pathogens. The following discussion considers key concepts in this respect.

### Insights from recent human *S. aureus* vaccine initiatives

Despite highly meritorious efforts, the development of a licensed, efficacious vaccine or immunotherapy targeting *S. aureus* has been elusive to date (see above). Multiple variables likely contribute to this situation, reflecting the complex host–pathogen relationship between *S. aureus* and human beings. Immunological insights from recent carefully designed vaccine clinical trials may aid in considering potentially improved strategies (Table 3). It should also be emphasized that even though efforts to-date have not yielded a licensed *S. aureus* vaccine, they have greatly contributed to our understanding of basic pathogenesis strategies of this pathogen, and the human immune responses to it. These advances cannot be overstated in terms of their positive contributions to the field and insights into design and development of efficacious vaccines.

**Table 3:** **Comparison of recent clinical trials of active vaccines and passive antibodies targeting *S. aureus* disease**.

Developer	Vaccine strategy	Target antigen(s)	Target patient(s)	Immunology	Outcome
Nabi	Bivalent polysaccharide-protein conjugate vaccine	Capsular polysaccharide types 5 and 8	Chronic hemodialysis patients	Robust antibody response; durable for greaterthan year	Phase 3: trended but did not statistically reduce incidence of invasive infection (93)
Inhibitex	Passive antibody (no adjuvant)	Clumping factor A (*ClfA*)	Established staphylococcemia; neonatal staphylococcemia	Rapid, durable systemic levels of α−ClfA antibody	Phase 3: trended but did not statistically reduce risk of developing invasive infection (111)
Merck	Recombinant protein with or without aluminum hydroxide	Iron surface determinant B (IsdB) siderophore	Cardiothoracic sternotomy patients	Robust antibody response	Phase 2: vaccine potentially associated with serious post-operative complications (89)

A prominent trend among the approaches exemplified above is an emphasis on robust antibody responses to the target antigen. While it is likely a contributing factor to human protection against the pathogen (43, 112–115), induction of humoral immunity alone may be insufficient to prevent *S. aureus* infections (116–118). Moreover, the observed efficacy of some vaccines in animal models of *S. aureus* infection may not entirely recapitulate human responses for several reasons. First, animal models often use adjuvants that are not applicable for use in humans, such as Complete Freund’s adjuvant (CFA). Such adjuvants may enhance immunity in animals that cannot be generated in humans using other adjuvants. Second, non-human mammals may defend against *S. aureus* via mechanisms that are not identical to human immunity (119–122). However, recent studies suggest that *S. aureus* organisms respond to human and other mammalian hosts (e.g., mice) in a nearly identical manner, suggesting consistent targets for vaccines and immune therapies (123, 124). Beyond these considerations, uncertainties in immune defenses, as well as fundamental host-pathogen relationships regarding normal flora status, may present special challenges to the development of an efficacious vaccine targeting *S. aureus*.

Thus, the experience to date in the development of vaccines targeting native *S. aureus* immunogens either in single or multi-antigen formats has provided only limited success. Even so, important new information has been gained from the many laudable efforts that have been made toward the development of *S. aureus* vaccines and immunotherapies (Tables 1–3). From these perspectives, innovative strategies such as vaccines applying convergent immunity that capitalize on emerging insights into efficacious host defense against this organism are needed.

### Historical insights into convergent immunity in human vaccines

In the context of vaccine development, “natural” immunity can be defined as inducing an immune response to an antigen that is naturally present in or on the target pathogen. This is the fundamental basis of vaccine development for most of the 200-year history of modern vaccine use, which typically reflects the principles of homologous and/or heterologous immunity (see above). Moreover, aspects of convergent immunity have been important factors in past and present vaccine development. Strategic use of an antigen that is convergent in structure and/or function to that in a target pathogen intends the outcome of altering and enhancing the protective efficacy of the vaccine beyond what can be obtained using the “natural” antigen. This strategy encompasses the use of epitope homologs that can effectively serve as surrogates of those in the target antigen, even though the origins of such homologs may be unrelated to the target pathogen. Specific examples follow, and some of these concepts have also been reviewed elsewhere (109, 125).

#### Cross-protection between related viral pathogens

Early human trials to develop a vaccine against smallpox were in fact based on the cowpox virus (126). It was similar enough to the human smallpox virus that the immune response to the cowpox virus was sufficient to protect from disease due to the smallpox virus. This is an early example of using convergent immunity to successfully defend against a target pathogen.

#### Toxoid antigens

A critical evolution in the development of vaccines against specific bacterial pathogens, in particular *Corynebacterium diphtheria* and *Clostridium tetani*, was the recognition that disease due to critical toxin(s) could be ameliorated by formaldehyde-treatment or “toxoiding” of a native toxin protein [see Ref. (127)], This process also allows the purified toxoid protein to be used as a direct immunogen rather than having to use antiserum from toxin-tolerized animals. Though the main goal of toxoids has been to abrogate toxin activity, a side-result has been to also create convergent immunogens successful in generating a protective immune response against the target organism.

#### Attenuated live virus vaccines

Since the early days of viral vaccine development, attenuation of the virulence of the target viral pathogen has been successfully employed to develop vaccines. Polio, measles, mumps, rubella, and varicella zoster are perhaps the most common examples of attenuated viruses used in human vaccines. Though the specific target of the attenuation may not be fully defined, these biological alterations, induced by imposing selection pressures on the virus under non-natural conditions in non-human viral propagation systems. These strategies include propagation in cells lines in which the virus had little or no pathogenic impact or reduced replication temperature, and are often directed to components of viral replication or intercellular translocation machinery, or to key viral immunogens responsible for protective immunity. Live viral vaccines are often attenuated by mutations favored during replication in non-natural hosts (e.g., influenza in embryonated chicken eggs). These permanent mutations make the attenuated viral antigen(s) distinct from those spread through human-to-human transmission. Ultimately, attenuated live virus vaccines ameliorate disease by interfering with the initiation or progression of target viral pathogenesis using immunogens and/or inducing immune responses not normally observed in nature. In addition, such approaches often aid in understanding pathogenesis of the target viral pathogen, and enhancement of immunogenicity to advance novel and more efficacious vaccines. Similar strategies of attenuation have also been used in some live bacterial vaccines, such as the BCG, typhoid, and typhus vaccines.

#### Consensus sequences in multi-clade HIV vaccines

Early in the efforts to develop a vaccine that would prevent HIV/AIDS, it was recognized that there were many clades of HIV and sequence variants of immunogens within each clade. One solution that has been attempted is the generation of “consensus sequence” protein antigens that would not align exactly with any one viral clade, but perhaps represent many or all [e.g., Ref. (128, 129)]. Though a clever concept, this approach has not yet resulted in protection against HIV disease in clinical trials required for evaluation. It remains to be seen whether such approaches will outpace more conventional strategies and lead to the efficacy and successful licensing of HIV/AIDS vaccines. That achievement will set the stage to evaluate the utility of natural versus convergent immunity in this vaccine target. In more recent times, this same concept is being adopted in the development of new influenza vaccines (130).

#### Use of recombinant protein antigens

Recombinant proteins can, but may not, mirror the composition and higher-order structure of their native template counterparts (see Designing Convergent Immunogens, below). Even though recombinant protein immunogens are intended to mimic a specific native protein, there are often modifications associated with the expression construct and/or manufacturing process to enhance productivity, optimize folding, reduce aggregation, or influence other molecular features. Such changes, the result of intentional or unintentional modifications in the new protein antigen, can produce antigens that are structurally and/or immunologically distinct from those found in the native antigen in the target pathogen. A simple example is found in the *Candida albicans* agglutinin-like sequence 3 (Als3) protein, which contains a six-His affinity tag to facilitate purification, yet is successful in stimulating a robust immune response in humans, including those who have been primed by natural exposure to *C. albicans* (81). However, as detailed below, because of intentional truncation and expression in a heterologous organism (rendering a non-native structure and glycosylation pattern), the Als3 antigen is no longer native to *C. albicans*. Thus, beyond modifications of a native antigen, using such an immunogen to protect against an organism (e.g., *S. aureus*) other than its original source by exploiting convergent antigen(s) of the target pathogen illustrates one application of convergent immunity, i.e., cross-kingdom convergent immunity.

#### Cross-protection of polysaccharide serotypes in pneumococcal vaccines

The concept of convergent immunogens is not limited to protein antigens. A parallel approach is well established for bacterial capsular polysaccharide antigens. For example, there is some evidence indicating that serotype 6B conjugate vaccine can cross-protect against serotype 6A (131) and that serotype 9V conjugate vaccine can be effective in protecting against serotype 9A infection (132). However, cross-protection between serotypes within the same pneumococcal serogroup remains controversial. Likewise, previous speculation that serotype 19F conjugate vaccine can provide cross-protection against serotype 19A is still controversial (133, 134), with epidemiological data suggesting that the inclusion of a serotype 19A conjugate component in the second-generation pneumococcal conjugate vaccine is beneficial (135).

#### Deleterious immunity

Deleterious immunological consequences may also occur due to unintended alterations of key immunogenic epitopes, as in the development of a vaccine targeting respiratory syncytial virus (RSV). In one case, a formalin-inactivated viral vaccine was formulated with an aluminum hydroxide suspension, commonly used as an adjuvant for bacterial vaccines. This vaccine, evaluated in infants and children, led to an immune response that actually exacerbated the viral infection rather than mitigated it; 80% of trial participants had to be hospitalized and two infants died (136). The specific cause of this vaccine failure is still debated, but further work on this important vaccine target was delayed by more than 30 years before new vaccine candidates were pursued in clinical trials.

Central dogma of conventional vaccinology is to use native antigen(s) from the target organism to induce protective immune responses against that organism. By comparison, convergent immunity strategically leverages non-native antigen homologs (including from organisms other than the target pathogen) to drive antigen-restricted immune responses against a target organism. The above examples illustrate how convergent immunity applies non-native antigen homologs to induce vaccine efficacy. Variations of this theme have been employed throughout the history of human vaccine development. Many attenuated live virus, polysaccharide, and recombinant protein vaccines, where no intentional manipulation of native antigen primary structure or composition has occurred, can be considered native antigens. Further, the increasing use of chemical modification of antigens (e.g., toxoids, conjugation, etc.) or protein sequence alteration (e.g., fusion proteins or truncated protein antigens) in licensed vaccines shows the successful application of customized native antigens in human vaccines. However, convergent immunity takes this concept one step further: use of a native or customized native antigen relative to one pathogen, and apply it to antigen-restricted protection targeting a different pathogen containing structurally and/or functionally homologous antigen(s). Thus, native immunity and convergent immunity are distinct but complementary forces in the design of efficacious vaccines. With advances in technology and understanding of protective immune responses, convergent immunity will almost certainly be leveraged in some form for future vaccine development, including for development of vaccines targeting *S. aureus*.

### Insights from use of convergent antigens in *S. aureus* vaccine studies

In 2008, it was shown that a recombinant protein based on the N-terminal region of a surface antigen, agglutinin-like sequence 3 protein from *C. albicans* (rAls3), which had previously been shown to be an adhesin (137) and an invasin (138), could protect mice from intravenous challenge with *S. aureus* (41). This predicted outcome was based on the discovery that Als3 had a high degree of structural homology with two surface proteins on *S. aureus*, clumping factor A (Clfa) and collagen-binding protein (Cna) (139). Moreover, the N-terminus of Als3 and other Als family members have close structural homology to adhesins and invasins from a number of microbial pathogens, including *Yersinia* spp. and other Gram-negative organisms, and hemagglutinin from influenza virus strains. Studies using rAls3 formulated with aluminum hydroxide (referred to as NDV-3; see below) and have now established a strong foundation for pursuing this antigen as an example of applying convergent immunity to contribute to the development of a successful *S. aureus* vaccine.

#### Murine bacteremia models

Prior studies from our group have demonstrated that immunization with the *C. albicans* rAls3 antigen induces protective efficacy against MRSA bacteremia in Balb/C mice (41). Further, in the specific conditions of this model, immunization with rAls3 evokes protective efficacy against hematogenously disseminated *S. aureus* infection in B cell but not T-cell knockout mice. Supporting a key role for T cells, non-immunized animals were protected by adoptive transfer of CD4+ T cells from NDV-3 immunized mice but not by transfer of B220+ B cells or serum from NDV-3 immunized mice. Further, NDV-3 protection against MRSA bacteremia was abrogated in IL-17A null mice (140). Collectively, this body of evidence is consistent with the emerging immunobiology of natural *S. aureus* antigens, in which Th17 pathway constituents are involved in defense against *S. aureus* and *C. albicans* in mice.

#### Murine skin/skin structure infection models

Recently, we evaluated the efficacy and immunological mechanisms of NDV-3 in mouse SSSI due to MRSA (141). Compared to adjuvant alone, NDV-3 immunization reduced abscess progression, severity, and MRSA density in skin following challenge by a variety of strains. Moreover, vaccination by NDV-3 mitigated hematogenous dissemination to kidneys. Corresponding to this, protective efficacy NDV-3 induced increases in CD3+ T-cell and neutrophil infiltration, and IL-17A, IL-22, and host-defense peptide expression in local settings of SSSI abscesses. These novel findings demonstrate that NDV-3 efficacy against MRSA in SSSI involves a robust and complementary response integrating innate and adaptive immune mechanisms.

#### Bovine mastitis models

Important insights regarding convergent antigen targeting *S. aureus* have also derived from recent efforts to control bovine mastitis. For example, Festa et al. used the domesticated tobacco plant (*Nicotiana tabacum*) to express the *S. aureus* virulence factor extracellular fibrinogen binding protein (Efb) as a vaccine immunogen (142). Expression of this bacterial antigen in the context of plant mechanisms of protein synthesis yields an immunogen that is homologous to the natural antigen. Mice orally immunized with this product have antigen-specific immune responses, which are likely discernable from those induced by the native bacterial antigen. The immune responses to native antigens, such as *S. aureus* whole cell and lysate preparations, have also been modified by novel means such as formulation with the ISCOM matrix adjuvant (143). This latter adjuvant has been reported to induce higher levels of specific antibody, as compared to aluminum hydroxide adjuvant (144). This suggests that an adjuvant can induce/enhance a non-natural response to a natural antigen. More detailed studies are needed to characterize the similarities and differences in immune response to this and other convergent antigens, as compared to those observed using the corresponding native antigens.

#### Human vaccination

Evidence for human immune responses to NDV-3 that parallel those in the above mouse models come from human clinical trials evaluating the safety, tolerability, and immunogenicity of NDV-3 versus saline placebo (81). NDV-3 formulations containing either 30 or 300 μg rAls3 were safe, well-tolerated, and achieved 100% seroconversion. Peak anti-rAls3 IgG_total_ and IgA_1_ antibody levels were detected within 14 days of receiving a single dose of vaccine. Moreover, a single dose of NDV-3 resulted in IL-17A and IFN-γ production from Als3 peptide-stimulated peripheral blood mononuclear cells (PBMCs) in the majority of vaccinated subjects within 7–28 days of vaccination. Seven days following a second dose of vaccine at 6 months post-initial vaccination, a modest increase was seen in anti-rAls3 IgG_total_ and IgA_1_, essentially recapitulating the first dose antibody response. Importantly, after the second dose, rAls3-specific IFN-γ-producing PBMCs were found in 100% of subjects and Als3-specific IL-17A-producing PBMCs were seen in 50–80% of subjects. Such responses are consistent with the expectation that humans are naturally exposed only to the native Als3 antigen as a consequence of ubiquitous exposure to *C. albicans*. The robust and durable response to NDV-3 immunization indicates rapid T- and B-cell recognition and response to the NDV-3 immunogen, and an enduring anamnestic response in human subjects. Of special note, the immunological determinants corresponding to protective efficacy elicited by the convergent antigen in NDV-3 in murine models of invasive staphylococcal infection and SSSI (high anti-rAls3 antibody titers and Th17 responses) are also observed in the human immune response to NDV-3. Thus, the present findings support the potential for NDV-3 to prevent or mitigate severity of bacteremia and/or SSSI due to MRSA or MSSA strains in humans. Taken together, this body of evidence supports the continued clinical evaluation of NDV-3 as a vaccine candidate to protect against disease caused by *S. aureus* and *Candida*.

It should also be noted that much remains to be learned with respect to the critical difference between non-protective and protective immune responses relative to *S. aureus*. For example, among the interesting evidence emerging from the field is the finding that human antibody generated in response to the rAls3 antigen appears to enhance human neutrophil opsonophagocytosis of target pathogens (145). In contrast, initial findings from B-cell knock out mouse models of bacteremic *S. aureus* infection were interpreted to suggest that B cells, and therefore antibody, may have lesser roles in host defense against systemic *S. aureus* infection in mice (41). The limits of these prior studies should be recognized and not preclude the potential importance of human antibody in optimal host defense against SSSI or bacteremia caused by *S. aureus* in humans. For example, the importance of subclass switching, acute versus anamnestic responses, as well as anatomically targeted antibody (e.g., mucosal IgA versus circulating IgG) remain to be understood with respect to immunity to *S. aureus*, particularly as regards this convergent vaccine antigen.

### Strategies for convergent vaccine antigens targeting *S. aureus*

The considerations detailed above provide an integrated framework for exploring novel convergent vaccine antigens targeting *S. aureus*. In effect, there may be other vaccine antigens that have one or more convergent features related to this target pathogen. These features may be due, if for no other reasons, to natural strain-to-strain variations of indigenous pathogens, and the collateral impacts of antigen production processes. However, potentially more transformative or paradigm-shifting approaches may be applied to develop convergent vaccine antigens that are specifically designed to induce protective immunity for *S. aureus* indications.

The remainder of this article focuses on strategies for designing and producing such antigens to induce convergent immunity for protection against disease due to *S. aureus*. The attractiveness of exploring convergent immunity in this respect is enhanced by a lack of success to date with conventional *S. aureus*-derived antigens. Collectively, these perspectives suggest that novel strategies will be required to address the challenge and increasing impact of multi-drug-resistant *S. aureus* infections with respect to public health. Although a multi-valent vaccine approach using native antigens may eventually prove successful, applying convergent immunity strategies is anticipated to accelerate or afford unique advantages in developing efficacious *S. aureus* vaccines or immunotherapies. This view is supported by the historical facts mentioned above and perspectives considered below.

#### Parallels in host-pathogen evolution

Divergent microorganisms, even across kingdoms, may develop identical or highly similar mechanisms to exploit shared targets or strategies in human hosts. This relationship is illustrated through the convergent structural, functional, and immunological determinants that transcend biological kingdoms in the fungus *Candida albicans* and the bacterium *Staphylococcus aureus*:

- *Candida* and *Staphylococcus* occupy similar anatomical niches in humans (epidermal/mucosal barriers)
- Both organisms have evolved similar pathogenesis strategies in humans (mucocutaneous colonization/opportunism/immune evasion/hematogenous dissemination)
- These organisms appear to share evolutionary parallels in structural biology [e.g., Als/microbial surface components recognizing adhesive matrix molecules (MSCRAMMs) are members of the immunoglobulin superfamily]
- Immunoglobulin superfamily proteins are functional homologs (e.g., Als3/ClfA and other homologous adhesins and invasins)
- *C. albicans* and *S. aureus* interactions appear to synergize in pathogenesis [e.g., Ref. (85, 146)]
- Immune defenses to both pathogens are alike, relying primarily upon host-defense peptides, induction of Th1 and Th17 pathways, and phagocyte-mediated responses that are likely potentiated by humoral and complement functions.

It follows that the human host almost certainly employs similar immune pathways and mechanisms to control these pathogens. Such relationships afford opportunities to develop innovative immunogenic agents and strategies including broad-spectrum vaccines and convergent immunogens that protect against diseases due to opportunistic pathogens such as *S. aureus* that are also normal flora.

#### Cross-kingdom convergent immunogens: *Candida* and *Staphylococcus*

**Figure 2:**
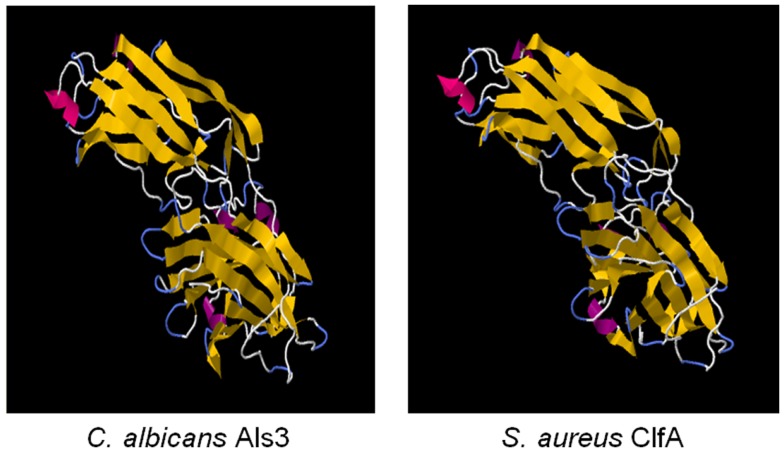
**The cross-kingdom immunogen rAls3 based on structure/function homology is shown**. Illustrated here are structurally homologous regions in Als3 (*C. albicans* adhesin/invasin; model) and ClfA proteins (*S. aureus* adhesin; deterministic structure analysis). The structural model of the Als3 N-terminus was generated as previously described using sequence threading and molecular dynamics [**left**; (130, 131)]. The cognate region in a homolog from *S. aureus* (clumping factor A; ClfA; **right**) is shown for comparison. Both proteins are members of the immunoglobulin superfamily and known virulence determinants. Note the overall highly similar three-dimensional β-barrel domains (gold arrows), interposed by hinge regions.

**Figure 3:**
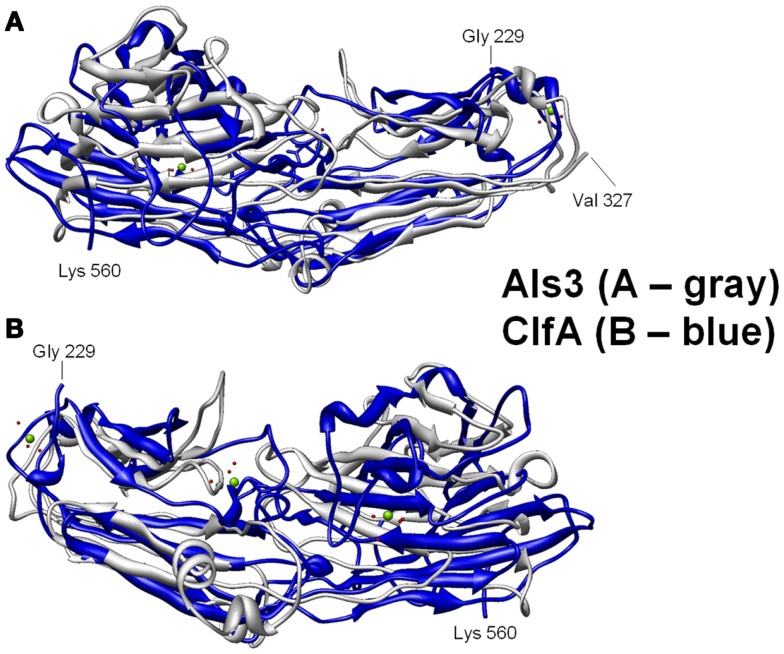
**Structural basis for B-cell epitopes shared in Als3 and ClfA is shown**. Combinatorial extension analysis was used to compare the model of Als3 with that of the known structure of ClfA. **(A)** Front view of the 3-D structural superimposition of homologous structural domains of Als3 (*Candida albicans*; gray) and ClfA (*Staphylococcus aureus*; blue); **(B)** rear view of the same region in protein homologs. The discovery, prediction, and validation of T-cell epitopes in such homologs offers further promise in design of innovative convergent vaccine antigens that induce efficacious cell-mediated immunity. Such striking convergence of linear (T cell) and 3-D (B-cell) epitopes in proteins with analogous functional and immunoprotective properties is consistent with the observation that *C. albicans* and *S. aureus* occupy very similar anatomic niches. It follows that humans have likely evolved common pathways of host defense against both pathogens, which may be targeted in novel vaccines that leverage convergent immunity.

We first identified the structural and functional homology among Als proteins of *Candida albicans*, and microbial surface components of *Staphylococcus* and other pathogens using computational bioinformatics and molecular modeling (139). Using 1° sequence homology threading and 3-D structural analysis, we found conservation of key sequence motifs and overall conformational homology among Als family proteins. Interestingly, hypervariable regions in sequences of these family members corresponded to diversity among extracellular loop regions. Next, we made the discovery that the N-terminal domains of Als1 and Als3 share unforeseen structural convergence with corresponding regions in *S. aureus* MSCRAMMS considered virulence factors, including clumping factor A (ClfA) (Figures 2 and 3; Table 4) (139). Similar to Als3 from *Candida*, such proteins mediate adhesion of *S. aureus* to host tissues or cells during pathogenesis. Notably, because Als3 and the *S. aureus* proteins have relatively low overall sequence identity (≤23%), their convergent structures would likely have been missed by sequence alignments alone. Based on this exciting discovery, we predicted that the rAls3 antigen derived from *C. albicans* could also induce protective immunity against *S. aureus* (41). We have since demonstrated the rAls3 vaccine to have efficacy in mouse models of hematogenous *C. albicans infection* (140, 147), vulvovaginal candidiasis (148), disseminated *S. aureus* infection (140), and in SSSI due to MRSA (141). Of importance, as predicted we found that vaccination with rAls3 induced robust humoral and cell-mediated immunity in these mouse models, paralleling responses observed in humans.

**Table 4:** **Quantitative sequence and three-dimensional structure homology analysis of *C. albicans* Als3 and selected homologs from *S. aureus* (139)**.

Protein 1	Protein 2	SeqID (%)	SeqSIM (%)	RMSD	*Z* score
Als3 (model)	1N67 (ClfA)	9	19	4.82^a^	5.19^b^
Als3 (model)	1D2P (Cna)	5	23	5.58	3.29
1N67 (ClfA)	1D2P (Cna)	9	20	5.44	3.50

*^a^Significance threshold <5.0*.

*^b^Significance threshold <3.5*.

*ClfA, clumping factor A; Cna, collagen-binding protein*.

##### Homology among B-cell epitopes

B-cell receptors identify three-dimensional epitopes, and the rAls3 antigen induces a strong antibody response in mice, primates, and humans. Moreover, antibodies from humans vaccinated with rAls3 promote opsonophagocytic activity *in vitro* against *S. aureus* (145). Therefore, the Als3 and homologous *S. aureus* antigens contain common immunogenic 3-D epitopes that are recognized by B cells. We have evaluated the structural features of such epitopes using complementary approaches. First, immune sera from mice and humans immunized with rAls3 antigen cross react with structural and functional homologs in *C. albicans* and *S. aureus* (Ibrahim and Yeaman, communication). Second, 3-D computational threading demonstrated robust conformational homology between Als3 from *C. albicans* and its functional homologs, for example, clumping factor A (ClfA) and collagen-binding protein A (Cna) of *S. aureus* (Table 4), as well as related homologs from other human pathogens (139). Third, our combinatorial extension analyses revealed specific conformational epitopes that are consistent with observed cross-reactivity of rAls3 immune sera to *C. albicans* and *S. aureus* (Figure 3). Taken together, these findings support the concept that these pathogens share common 3-D epitopes capable of inducing cross-reactive antibodies to both organisms.

##### Homology among T-cell epitopes

Our observations also pointed to common T-cell epitopes in *C. albicans* and *S. aureus* antigens, including: (1) protective efficacy of rAls3 in animal models of *C. albicans* and *S. aureus* infection; (2) T-cell response profiles indicating that rAls3 induced robust T-cell responses in both mice and humans; (3) sera predominantly include IgG subclasses that rely on T cells for B-cell activation and antibody production; and (4) overall low sequence identity between Als family proteins of *Candida* and MSCRAMM proteins of *S. aureus* suggested that regions where sequence homology did exist would hold insights into T-cell epitope sequences. Because vaccination of mice with rAsl3 induced protection by mechanisms including T-cell responses, we applied bioinformatic mining and computational modeling to predict T-cell epitopes in rAls3. In this process, we predicted an immunodominant epitope to have the sequence WNFPVSSDSFSYT (patent pending). As predicted, this epitope is very similar to the immunodominant human T-cell epitope of Als3 as experimentally validated by Bar et al. (149). Further, this epitope was reported to have 10-fold greater MHC-II peptide-binding groove affinity (264 nM) as compared with any other T-cell epitope, supporting robust predictive accuracy of our immunogen discovery and design approach.

### Leveraging convergent immunity for innovative vaccines

As illustrated above, by discovering non-obvious parallels in host–pathogen relationships, and exploiting structurally and functionally convergent antigens, we have generated broad-spectrum vaccines, which induce protective immunity against multiple pathogens. Our work leverages three tenets: (1) host defenses protective against pathogens having antigenic or strategic parallels in pathogenesis may be elicited by convergent vaccine antigens; (2) bioinformatics integrated with computational systems modeling can reveal and optimize cryptic epitope and immunological homologies for use in designing convergent immunogens with efficacy against diverse pathogens; and (3) immune defenses may be optimized to afford protective efficacy in specific anatomic, physiological, immunological, and/or microbiological contexts. These concepts are consistent with innovative approaches, including convergent immunity, as illustrated below.

#### Designing convergent immunogens

Engineered changes are common in recombinant protein antigens. Small changes in the composition, sequence, and structure between a manufactured vaccine antigen and the native form of that antigen (e.g., amino acid substitution and/or truncations of proteins, modification of glycosylation, attenuation of pathogenic capacity, or replication), have the potential to alter protective immune responses. If such modifications are too moderate, these strategies are likely to be silent or undecipherable to the functional immune response. For purposes of this discussion, we are more concerned with larger changes that are expected to impose distinct differences from the native antigen. Fundamental to the design of antigens is the consideration of primary sequence, post-translational or production process-induced modifications, higher-order structure, and molecular size and/or aggregation state.

##### Linear sequence epitopes

T lymphocytes detect linear peptide sequence epitopes presented by APC in the context of MHC class I (e.g., HLA A, B, or C; CD8+ T cells) or MHC class II (e.g., HLA DP, DQ, DR; CD4+ T cells). Thus, vaccines that induce efficacious T-cell responses rely upon the appropriate presentation of primary sequence epitopes and ensuing polarization to govern appropriate and protective immune response paradigms (e.g., Th1, Th2, Th17). As detailed above, vaccines that appear to induce efficacious responses in experimental models, and those which are immunogenic in humans, induce robust biomarkers of T-cell response. For example, IFN-γ and IL-17A are involved in efficacious vaccine responses in mouse models of MRSA bacteremia (41, 140) and SSSI (141) due to *S. aureus*, and these same immune response profiles are observed in humans immunized with this vaccine candidate (81). A key to design of efficacious vaccines targeting *S. aureus* is the understanding of biomarkers that offer the greatest insight into protective efficacy. For example, because IFN-γ and IL-17A counter-modulate one another, vaccines that promote maximal T- cell induction of such cytokines may not confer maximal protection. Thus, the identification of optimal T cell-mediated immune signatures, rather than maximal responses, may provide new opportunities to develop vaccines targeting *S. aureus*. Moreover, it remains to be determined whether such biomarkers may be best sought from bloodstream or tissue-specific biological specimens (e.g., mucosa, lymph node, other). In addition to design of cognate T-cell epitopes from pathogens themselves, engineering flanking or other common sequence motifs known to stimulate T cells and perhaps polarize to a given paradigm (e.g., Th17 versus Th1) is emerging in the design of vaccines that induce cell-mediated immunity (150).

##### Higher-order structures

The importance of T-cell responses in designing efficacious vaccines has received increased attention with respect to linear immune epitopes. However, large, conformational antigens designed to induce convergent immune responses also have emerging promise in the generation of novel efficacious vaccines. In this respect, a non-exhaustive list of examples might include the following.

###### 3-D structural homologs

As mentioned above, antibody induction has been a mainstay of immunization strategies classically. However, exemplified in the HIV experience and more recently with *S. aureus*, antibody alone may not prove to be the optimally protective immune response. However, there is important crosstalk that occurs between antibody and other immune effector systems (e.g., phagocytes, complement, etc.). Thus, vaccines that evoke convergent humoral immunity could be designed to optimize the most efficacious functional type of antibodies. For example, human IgG_1_ and IgG_3_ subclasses typically interact with Fcγ receptors on professional phagocytes most efficiently. Therefore, enhancing protective phagocytic defenses against *S. aureus* could involve design of convergent immunogens having 3-D structures that evoke high target-affinity Fab domains, and also promote subclass switching as above to optimize Fc-mediated opsonic functions. By comparison, protective immunity requiring complement fixation may best promote induction of antigen-restricted IgM and IgG_2_ antibody subclasses, for example. Likewise, vaccines intended to protect against colonization or infection of mucosal surfaces may be optimized to induce secretory IgA subclasses. Through each of these examples, convergent 3-D or conformational immunogens provide opportunities to induce antibody profiles distinct from those induced by B-cell epitopes inherent to the native antigens themselves. Also important in this regard is a need for cytokine conditioning and T-cell signaling for B-cell activation and antibody generation in response to an antigen. Obviously, recent discoveries point to the importance of B cells as APC for T-cell activation. Such considerations further suggest critical immunogenic relationships between T- and B-cell epitopes needed in an optimally efficacious vaccine.

###### Fusion constructs

**Figure 4:**
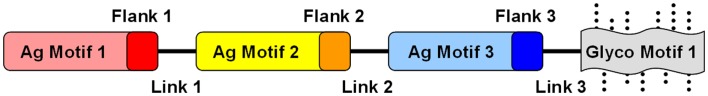
**Conceptual design of modular and combinatorial convergent vaccine immunogens**. Illustrated here is an example of a convergent vaccine immunogen that integrates multiple antigen motifs (*Ag motif*) containing flanking sequences (*Flank*) optimized as T-cell epitopes, interposed by strategic linker domains (*Link*) in a fusion construct. By virtue of its expression in a strategic heterologous system, glycosylation motifs (*Glyco motif*) or lack thereof would be favored to stimulate or target immune pattern recognition receptors (PRRs) to evoke optimal responses for protective efficacy.

Fusion protein constructs have also been explored as a means of creating efficient “designer” antigens. This approach has the advantage that multiple epitopes can be combined into a smaller number of recombinant proteins, assuming that the essential features of the epitopes can be conserved in the resulting protein product (Figure 4). A particularly extreme example of a fusion protein antigen appears to be the Group A *Streptococcus* (GAS) vaccine originally developed by ID Biomedical, which consisted of multiple recombinant proteins, each of which was a fusion of immunogenic regions from a given surface protein from a total of 26 serotypes of GAS (151).

Fusion constructs may contain structural elements already demonstrated to be determinants of protective efficacy. In addition to the known virulence antigens (or immunogenic domains thereof) that contain fundamental B- and T-cell motifs, fusion proteins can be further designed to encompass antigens to enhance mechanisms of efficacy against the target organism(s) (Figure 4). Immunogenic modules can be interposed by linker domains that enable high-fidelity presentation of the 3-D structure of each antigen to be maintained (152). Such strategies can also facilitate appropriate processing of the fusion protein (e.g., proteolysis and presentation of linear epitopes) by APC for T-cell receptors. Such modular components will likely impose conformational influences on one another, and can be rearranged to optimize protective effects based on *in vivo* efficacy and immune response. Furthermore, such constructs can be engineered to contain sequences that enhance antigen-presenting and intrinsic adjuvant effects that promote ideal polarization of the immune system to the target pathogen.

###### Antigen context

**Figure 5:**
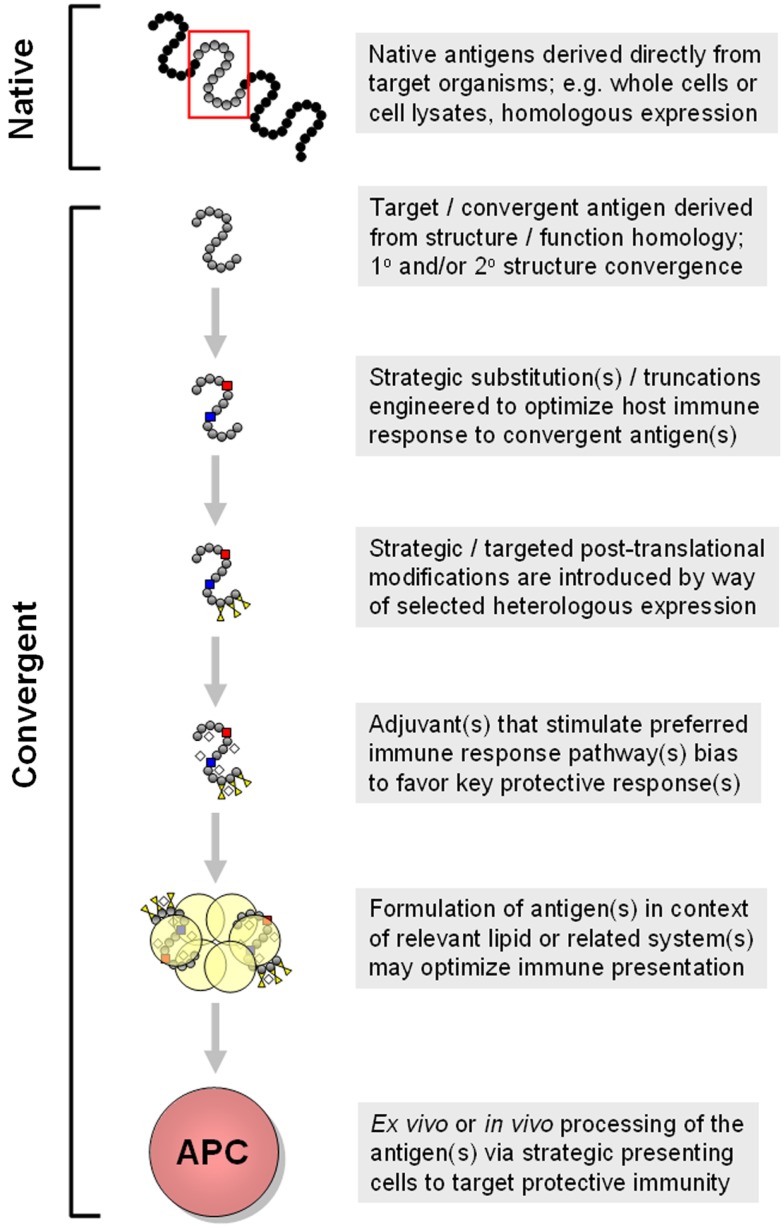
**Progressive modes for generation of convergent immunogens are shown**. In this example, multiple concepts detailed in the text are shown in a sequential manner that yields increasingly strategic convergent immunogens. Approaches such as these are being employed in design of vaccines to elicit optimally protective immune responses that include tissue-targeted and cross-kingdom mechanisms of action. These and related aspects of convergent immunity may be necessary for efficacious vaccines targeting opportunistic pathogens such as *S. aureus*.

Traditionally, many vaccines have been viewed as protein-based immunogens in solution, functioning individually to prompt specific responses targeting the cognate antigen(s). Beyond this view, interactions of antigens, or antigens organized in macromolecular systems or nanoparticles, afford additional strategies for beneficial impacts of convergent immunity (153, 154). For example, a protein in solution may have very different conformational epitopes as compared to the identical protein in context of a lipid bilayer. As many vaccine antigens derive from membrane-associated virulence factors, solution-based immunogens may miss important opportunities to induce protective immunity. Likewise, distinct antigens may interact with one another in a vaccine suspension, creating second or greater order epitopes for immune exposure. From these examples, it is reasonable to anticipate that novel vaccines targeting *S. aureus* may contain one or more antigens in conditional states. Development of liposomal or nanoparticle vaccines allow some immunogens to be presented in context of biological membrane systems (Figure 5). Such strategies offer advantages in presenting epitopes so as to engender optimally protective, rather than maximally quantitative, B-cell responses.

###### Targeted antigen presentation

It is increasingly apparent that the human immune system detects immunogenic determinants in specific contexts that bias specific immune responses. For example, in mammalian lung mucosa, resident dendritic cells (e.g., class I; MHCII^hi^/CD11b^+^/CD11c^+^) appear to detect, process, and traffic antigens in a manner optimized for MHC class II presentation and ensuing activation of proximate stromal CD4^+^ T cells (155, 156). These functions are not identical in class II monocytic dendritic cells (e.g., IL-12 secreting), or class V dermal dendritic cells. As a result, immune responses from such distinct contexts of antigen processing, presentation, and trafficking can be very different. It follows that convergent immunity best suited to protect against *S. aureus* infections of specific tissue sites or anatomic contexts may necessarily need to be designed to engage APC most relevant to a given site. Further, specific vaccines designed to target antigen presentation to specific T and/or B-cell subsets is a logical extension of this strategy of convergent immunity (157). These examples illustrate the larger concepts that convergent vaccine antigens might be designed to comprise multi-component sets of core epitopes engineered to stimulate specific subsets of APC to evoke optimal host defenses for specific anatomic niche(s).

##### Post-translational or production process-induced modifications

Beyond the design of non-natural antigenic sequences or constructs themselves, the use of non-native hosts for their expression affords further dimensions in the generation of antigens for stimulation of convergent immunity. For example, purposeful substitutions, additions (e.g., six-His tags) and/or truncations are commonly employed to improve the productivity of expression cell lines or to minimize or eliminate aggregation of recombinant proteins. Such specifications may be integrated into various approaches for strategic heterologous expression of a given antigen for optimal induction of protective immunity. These strategies might also incorporate the choice of a heterologous host organism, as well as conditions under which an organism is induced to express an antigen. A non-exhaustive illustration of the spectrum of potential considerations in this regard might include strategic uses of *Escherichia coli, Saccharomyces cerevisiae, Pichia pastoris* non-human mammalian cell lines (e.g., Chinese hamster ovary cells), or human cell lines. Each has its advantages and drawbacks, including a greater theoretical potential to generate “humanized” antigens that, in concept, may have heightened risk of inducing autoimmune disease.

#### Combination vaccine products

Identification and development of convergent immunogens such as rAls3 for an *S. aureus* vaccine may also complement the use of native antigens and provide surrogate antigens to induce extended immunological response. Moreover, this approach represents a new category of antigen that may be considered in the context of a combination vaccine product. The choice of antigens to use in such a combination product is driven by considering multiple factors, including the role of the antigen in the biology of the organism (e.g., function; accessibility), the uniqueness of the immunogen with respect to human proteins or other microbial targets, the type of immune response induced by the antigen, or a combination of the above. To date, most multi-component vaccines used in clinical evaluations (see Table 2) have been driven more so by consideration of the roles of the antigens in the biology of the organism. In some cases, adjuvants are used to influence the type of immune response being brought to bear (see below). However, more recently, consideration is being directed to a mixed approach that also considers the relationship among antigens, and types of immune responses they induce with respect to *S. aureus* (118, 158, 159).

#### Adjuvants

The role of adjuvants in vaccines can be considered either as a way to direct the immune response to the vaccine antigens (e.g., use of aluminum hydroxide suspension to bias toward a Th2 response) or as a way to enhance immune responses to the immune stimuli inherent to a given antigen (e.g., inclusion of aluminum hydroxide does not appear to influence the T-cell response to rAls3). As such, the inclusion of an adjuvant in a formulation with a convergent antigen provides yet another opportunity to influence the immune responses to a vaccine. The real value of the adjuvant can only be determined by comparative studies in the target human population to: (1) establish an increase in efficacy with use of the adjuvant (versus without the adjuvant); or (2) by first establishing surrogate markers of protection and then observing the increase in those markers in the presence versus the absence of the adjuvant. While animal models may be useful in these respects, final evaluation in humans remains the only convincing way to establish the value of adding a given adjuvant to a vaccine formulation.

### Strategic immunization regimens

Convergent immunity may also benefit from thoughtful timing and modes of vaccine administration. With *S. aureus* being a commensal pathogen, it stands to reason that most if not all humans have been primed for *S. aureus* natural antigens by exposure to the bacterium. In this case, strategic vaccination regimens that may bias acute or more durable responses might be favored, depending on whether long-term or short-term immunization is the objective of vaccination. Long-term efficacy is necessary in protection against chronic threat of infection, whereas short-term protection may be sufficient when risk of infection can be anticipated (e.g., transplantation, chemotherapy, surgery, etc.). Short-term immunization may only require a single dose of vaccine, which in the presence of pre-existing immune response to the immunogen homolog, can be used to extend the scope of natural anamnestic response that enhance convergent immunity. As has been shown with the rAls3 antigen, the magnitude of this boost in both B- and T-cell responses may be moderated by the dose level of antigen administered (81). For vaccines containing a combination of antigens, the option for a single dose to induce a natural anamnestic response relies on each antigen having been primed by natural exposure to the pathogen. Longer-term immunity may require additional doses of vaccine, particularly if a strong T-cell response is needed. In this situation, doses may be given as a series of vaccinations over a short period of time to prompt a robust initial immune response, or given over longer time intervals (e.g., annually) to maintain an elevated state of immune readiness. However, it should be kept in mind that a maximal immune response may not be an optimal immune response, as the ultimate goal is not to induce immune response but to prevent or treat disease.

Beyond timing, it is increasingly evident that mode of vaccine administration can have significant impact on immune response. For example, as above, an identical immunogen that is administered via intra-muscular, subcutaneous, or even intra-dermal routes often induce distinctive immune response profiles. These considerations illustrate the larger concepts that convergent vaccine antigens might be designed to comprise multi-component sets of core antigens that have been engineered to stimulate specific subsets of dendritic or other APC in such a manner as to evoke collectively optimal host defenses for specific anatomic niche(s).

### Coordinate induction

Protective host defense versus *S. aureus* requires a highly coordinated and multi-focused immune response. An overarching strategy in convergent immunity is to develop vaccines or immunotherapeutics that bring complementary host defenses together to achieve protection against infection that could not be achieved by any single immune mechanism alone. This strategy addresses the reality that an infection caused by *S. aureus* contains organisms in different growth phases, that occupy different anatomic niches, and that may be refractory to one or more immune effectors alone. A conceptual example would be a multi-component vaccine, in which distinct immunogens are optimized to induce a Th17 response for neutrophil enhancement, Th2 response for induction of IgG_1_/IgG_3_ opsonophagocytic antibodies, and Th22 response to promote the induction of host-defense peptides most appropriate to act on the target pathogen in relevant tissues. Such strategies for coordinate induction may afford advantages in optimizing efficacy via inclusion of both natural and convergent immunity. It is possible if not likely that combinations of such strategies will be leveraged in design and evaluation of multi-valent vaccines targeting *S. aureus*. It may also be more realistic than not that distinct vaccines or immunotherapeutic modalities may be needed to protect against *S. aureus* infections of specific anatomic niches or compartments. Such vaccines would be optimized to marshal the optimally protective host defenses for a given niche. For example, vaccines designed to protect mucocutaneous tissues may induce distinct adaptive and effector mechanisms than those most efficacious in defending against *S. aureus* bacteremia. These concepts integrate the realties that the repertoire of immune targets and virulence strategies of this organism in distinct compartments are very different, as are immune responses best to defend against them.

## Prospectus

The application and optimization of convergent immunity derives from historical success in vaccine discovery and development. In this sense, vaccines that induce protective immunity in response to antigens, which are non-identical to those found in nature has many potential advantages in development of improved vaccines targeting *S. aureus* or other pathogens. Yet, much remains to be learned regarding the secrets by which the human immune system largely outwits *S. aureus* on a day-to-day basis. By unlocking these mechanisms, vaccines comprising convergent as well as natural antigens have the potential to address the public health concerns imposed by *S. aureus* and likely other pathogens as well.

As proposed herein, optimally efficacious vaccines and immunotherapeutics protective against *S. aureus* disease will likely rely upon:

- A more comprehensive understanding of the critical immunoprotective mechanisms targeting this organism, including those that may not be known or fully appreciated at present. A key to this concept is differentiating non-protective versus protective immune responses. It follows that specific biomarkers that prioritize protective immune responses over those, which are not protective are much needed. Included in these concepts are molecular and cellular effectors of immunity not traditionally considered to be active in defense against *S. aureus* (e.g., cell-mediated immunity; interactions between innate host-defense peptides and professional phagocytes, etc.). These effector mechanisms may be additive or synergistic interactions of immune responses, and/or not currently detected or prioritized in the evaluation of vaccines.
- Leveraging of convergent immunity in targeting normal flora organisms for which there may be pre-existing baseline immune responses.
- Innovative design and deployment of convergent antigens as surrogates of natural antigens that trigger protective immune responses, which optimize efficacy or promote immunity that would not otherwise be induced by recapitulation of native antigens.
- Immunization regimens and strategies that induce dynamic and coordinated immune responses that are optimized to anatomic, physiological, or microbiological contexts best suited to address *S. aureus* pathogenesis or immune subversion strategies.
- Use of strategic adjuvants to promote antigen-restricted immune responses that afford protection against the ultimate target pathogen(s), even if the antigen(s) themselves may not be native to these pathogens.
- Combinations of the above that employ strategic use of heterologous expression, modular antigen design, and emerging technologies to evoke optimally protective rather than maximally detectable immune responses, and advances in biomarker signatures to discern the difference; and
- Avoidance of untoward effects in dysregulated or dysfunctional immune responses to vaccine antigens, including autoimmune disease.

## Conflict of Interest Statement

The authors are founders, consultants, or employees of NovaDigm Therapeutics, Inc., which is developing novel vaccines targeting *Staphylococcus aureus* and other pathogens. Some of the authors are inventors on patent applications related to the use of *Candida albicans* Als3 as a vaccine antigen targeting *Candida* spp. and the cross-kingdom protection targeting *S. aureus*. NovaDigm has licensed the world-wide rights to this intellectual property.

## References

[1] David MZ Daum RS Community-associated methicillin-resistant *Staphylococcus aureus*: epidemiology and clinical consequences of an emerging epidemic. Clin Microbiol Rev (2010) 23(3):616–87 10.1128/CMR.00081-09 20610826 PMC2901661

[2] Gunderson CG Martinello RA A systematic review of bacteremias in cellulitis and erysipelas. J Infect (2012) 64(2):148–55 10.1016/j.jinf.2011.11.004 22101078

[3] DeLeo FR Otto M Kreiswirth BN Chambers HF Community-associated methicillin-resistant *Staphylococcus aureus*. Lancet (2010) 375(9725):1557–68 10.1016/S0140-6736(09)61999-1 20206987 PMC3511788

[4] Geria AN Schwartz RA Impetigo update: new challenges in the era of methicillin resistance. Cutis (2010) 85(2):65–70 20349679

[5] Karamatsu ML Thorp AW Brown L Changes in community-associated methicillin-resistant *Staphylococcus aureus* skin and soft tissue infections presenting to the pediatric emergency department: comparing 2003 to 2008. Pediatr Emerg Care (2012) 28(2):131–5 10.1097/PEC.0b013e318243fa36 22270497

[6] Hersh AL Chambers HF Maselli JH Gonzales R National trends in ambulatory visits and antibiotic prescribing for skin and soft-tissue infections. Arch Intern Med (2008) 168(14):1585–91 10.1001/archinte.168.14.1585 18663172

[7] Chambers HF Community-associated MRSA – resistance and virulence converge. N Engl J Med (2005) 352(14):1485–7 10.1056/NEJMe058023 15814886

[8] Dantes R Mu Y Belflower R Aragon D Dumyati G Harrison LH National burden of invasive methicillin-resistant *Staphylococcus aureus* infections, United States, 2011. JAMA Intern Med (2013) 173(21):1970–8 10.1001/jamainternmed.2013.10423 24043270 PMC10887428

[9] Tattevin P Schwartz BS Graber CJ Volinski J Bhukhen A Bhukhen A Concurrent epidemics of skin and soft tissue infection and bloodstream infection due to community-associated methicillin-resistant *Staphylococcus aureus*. Clin Infect Dis (2012) 55(6):781–8 10.1093/cid/cis527 22670044 PMC3657511

[10] Klevens RM Morrison MA Nadle J Petit S Gershman K Ray S Invasive methicillin-resistant *Staphylococcus aureus* infections in the United States. JAMA (2007) 298(15):1763–71 10.1001/jama.298.15.1763 17940231

[11] Song X Cogen J Singh N Incidence of methicillin-resistant *Staphylococcus aureus* infection in a children’s hospital in the Washington metropolitan area of the United States, 2003-2010. Emerg Microbes Infect (2013) 2(10):e69 10.1038/emi.2013.69 PMC3826068 26038439

[12] Chambers HF DeLeo FR Waves of resistance: *Staphylococcus aureus* in the antibiotic era. Nat Rev Microbiol (2009) 7:629–41 10.1038/nrmicro2200 19680247 PMC2871281

[13] Dryden MS Complicated skin and soft tissue infection. J Antimicrob Chemother (2010) 65(Suppl 3):iii35–44 10.1093/jac/dkq302 20876627

[14] Iwamoto M Mu Y Lynfield R Bulens SN Nadle J Aragon D Trends in invasive methicillin-resistant *Staphylococcus aureus* infections. Pediatrics (2013) 132(4):e817–24 10.1542/peds.2013-1112 24062373 PMC11931424

[15] Catry B Latour K Jans B Vandendriessche S Preal R Mertens K Risk factors for methicillin resistant *Staphylococcus aureus*: a multi-laboratory study. PLoS One (2014) 9(2):e89579 10.1371/journal.pone.0089579 24586887 PMC3935888

[16] Jiménez JN Ocampo AM Vanegas JM Rodriguez EA Mediavilla JR Chen L A comparison of methicillin-resistant and methicillin-susceptible *Staphylococcus aureus* reveals no clinical and epidemiological but molecular differences. Int J Med Microbiol (2013) 303(2):76–83 10.1016/j.ijmm.2012.12.003 23369303

[17] Stenehjem E Rimland D MRSA nasal colonization burden and risk of MRSA infection. Am J Infect Control (2013) 41(5):405–10 10.1016/j.ajic.2012.07.017 23261345 PMC3685139

[18] Quezada SM Steinberger EK Cross RK Association of age at diagnosis and Crohn’s disease phenotype. Age Ageing (2013) 42(1):102–6 10.1093/ageing/afs107 22918090 PMC3518904

[19] Duffy J Dumyati G Bulens S Namburi S Gellert A Fridkin SK Community-onset invasive methicillin-resistant *Staphylococcus aureus* infections following hospital discharge. Am J Infect Control (2013) 41(9):782–6 10.1016/j.ajic.2012.10.020 23394888 PMC12035715

[20] Datta R Shah A Huang SS Cui E Nguyen V Welbourne SJ High nasal burden of methicillin-resistant *Staphylococcus aureus* increases risk of invasive disease. J Clin Microbiol (2014) 52(1):312–4 10.1128/JCM.01606-13 24153126 PMC3911445

[21] Suresh R Mosser DM Pattern recognition receptors in innate immunity, host defense, and immunopathology. Adv Physiol Educ (2013) 37(4):284–91 10.1152/advan.00058.2013 24292903 PMC4089092

[22] Zhong Y Kinio A Saleh M Functions of NOD-like receptors in human diseases. Front Immunol (2013) 4:333 10.3389/fimmu.2013.00333 24137163 PMC3797414

[23] Bauer S Muller T Hamm S Pattern recognition by toll-like receptors. Adv Exp Med Biol (2009) 653:15–34 10.1007/978-1-4419-0901-5_2 19799109

[24] Yeaman MR Platelets: at the nexus of antimicrobial defence. Nat Rev Microbiol (2014) 12(6):426–37 10.1038/nrmicro3269 24830471

[25] Nestle FO Di Meglio P Qin JZ Nickoloff BJ Skin immune sentinels in health and disease. Nat Rev Immunol (2009) 9(10):679–91 10.1038/nri2622 19763149 PMC2947825

[26] Kupper TS Fuhlbrigge RC Immune surveillance in the skin: mechanisms and clinical consequences. Nat Rev Immunol (2004) 4(3):211–22 10.1038/nri1310 15039758 PMC7097017

[27] Renner ED Rylaarsdam S Anover-Sombke S Rack AL Reichenbach J Carey JC Novel signal transducer and activator of transcription 3 (STAT3) mutations, reduced T(H)17 cell numbers, and variably defective STAT3 phosphorylation in hyper-IgE syndrome. J Allergy Clin Immunol (2008) 122(1):181–7 10.1016/j.jaci.2008.04.037 18602572 PMC4560358

[28] Horváth R Rožková D Laštovička J Poločcková A Sedláček P Sedivá A Expansion of T helper type 17 lymphocytes in patients with chronic granulomatous disease. Clin Exp Immunol (2011) 166(1):26–33 10.1111/j.1365-2249.2011.04449.x 21910722 PMC3193916

[29] de Luca A Smeekens SP Casagrande A Iannitti R Conway KL Gresnigt MS IL-1 receptor blockade restores autophagy and reduces inflammation in chronic granulomatous disease in mice and in humans. Proc Natl Acad Sci U S A (2014) 111(9):3526–31 10.1073/pnas.1322831111 24550444 PMC3948220

[30] Miller LS Pietras EM Uricchio LH Hirano K Rao S Lin H Inflammasome-mediated production of IL-1beta is required for neutrophil recruitment against *Staphylococcus aureus* in vivo. J Immunol (2007) 179(10):6933–42 10.4049/jimmunol.179.10.6933 17982084

[31] Miller LS Cho JS Immunity against *Staphylococcus aureus* cutaneous infections. Nat Rev Immunol (2011) 11(8):505–18 10.1038/nri3010 21720387 PMC5868361

[32] Greenberg JA David MZ Hall JB Kress JP Immune dysfunction prior to *Staphylococcus aureus* bacteremia is a determinant of long-term mortality. PLoS One (2014) 9(2):e88197 10.1371/journal.pone.0088197 24505428 PMC3914899

[33] Metzger DW Sun K Immune dysfunction and bacterial coinfections following influenza. J Immunol (2013) 191(5):2047–52 10.4049/jimmunol.1301152 23964104 PMC3760235

[34] Cho JS Pietras EM Garcia NC Ramos RI Farzam DM Monroe HR IL-17 is essential for host defense against cutaneous *Staphylococcus aureus* infection in mice. J Clin Invest (2010) 120(5):1762–73 10.1172/JCI40891 20364087 PMC2860944

[35] Murphy AG O’Keeffe KM Lalor SJ Maher BM Mills KH McLoughlin RM *Staphylococcus aureus* infection of mice expands a population of memory gammadelta T cells that are protective against subsequent infection. J Immunol (2014) 192(8):3697–708 10.4049/jimmunol.1303420 24623128 PMC3979672

[36] Krishna S Miller LS Host-pathogen interactions between the skin and *Staphylococcus aureus*. Curr Opin Microbiol (2012) 15(1):28–35 10.1016/j.mib.2011.11.003 22137885 PMC3265682

[37] Bergstresser PR Tigelaar RE Dees JH Streilein JW Thy-1 antigen-bearing dendritic cells populate murine epidermis. J Invest Dermatol (1983) 81(3):286–8 10.1111/1523-1747.ep12518332 6136547

[38] McLoughlin RM Solinga RM Rich J Zaleski KJ Cocchiaro JL Risley A CD4+ T cells and CXC chemokines modulate the pathogenesis of *Staphylococcus aureus* wound infections. Proc Natl Acad Sci U S A (2006) 103(27):10408–13 10.1073/pnas.0508961103 16801559 PMC1502471

[39] Mölne L Corthay A Holmdahl R Tarkowski A Role of gamma/delta T cell receptor-expressing lymphocytes in cutaneous infection caused by *Staphylococcus aureus*. Clin Exp Immunol (2003) 132(2):209–15 10.1046/j.1365-2249.2003.02151.x 12699407 PMC1808706

[40] Ishigame H Kakuta S Nagai T Kadoki M Nambu A Komiyama Y Differential roles of interleukin-17A and -17F in host defense against mucoepithelial bacterial infection and allergic responses. Immunity (2009) 30(1):108–19 10.1016/j.immuni.2008.11.009 19144317

[41] Spellberg B Ibrahim AS Yeaman MR Lin L Fu Y Avanesian V The antifungal vaccine derived from the recombinant N terminus of Als3p protects mice against the bacterium *Staphylococcus aureus*. Infect Immun (2008) 76(10):4574–80 10.1128/IAI.00700-08 18644876 PMC2546811

[42] Cho JS Zussman J Donegan NP Ramos RI Garcia NC Uslan DZ Noninvasive in vivo imaging to evaluate immune responses and antimicrobial therapy against *Staphylococcus aureus* and USA300 MRSA skin infections. J Invest Dermatol (2011) 131(4):907–15 10.1038/jid.2010.417 21191403 PMC3059384

[43] Montgomery CP Daniels M Zhao F Alegre ML Chong AS Daum RS Protective immunity against recurrent *Staphylococcus aureus* skin infection requires antibody and interleukin-17A. Infect Immun (2014) 82(5):2125–34 10.1128/IAI.01491-14 24614654 PMC3993461

[44] Zeng WP “All things considered”: transcriptional regulation of T helper type 2 cell differentiation from precursor to effector activation. Immunology (2013) 140(1):31–8 10.1111/imm.12121 23668241 PMC3809703

[45] Conlan S Kong HH Segre JA Species-level analysis of DNA sequence data from the NIH Human Microbiome Project. PLoS One (2012) 7(10):e47075 10.1371/journal.pone.0047075 23071716 PMC3468466

[46] Fischer CL Drake DR Dawson DV Blanchette DR Brogden KA Wertz PW Antibacterial activity of sphingoid bases and fatty acids against Gram-positive and Gram-negative bacteria. Antimicrob Agents Chemother (2012) 56(3):1157–61 10.1128/AAC.05151-11 22155833 PMC3294957

[47] Wille JJ Kydonieus A Palmitoleic acid isomer (C16:1delta6) in human skin sebum is effective against gram-positive bacteria. Skin Pharmacol Appl Skin Physiol (2003) 16(3):176–87 10.1159/000069757 12677098

[48] Yount NY Yeaman MR Emerging themes and therapeutic prospects for anti-infective peptides. Annu Rev Pharmacol Toxicol (2012) 52:337–60 10.1146/annurev-pharmtox-010611-134535 22235859

[49] Yeaman MR Yount NY Unifying themes in host defence effector polypeptides. Nat Rev Microbiol (2007) 5(9):727–40 10.1038/nrmicro1744 17703227

[50] Yeaman MR Yount NY Mechanisms of antimicrobial peptide action and resistance. Pharmacol Rev (2003) 55(1):27–55 10.1124/pr.55.1.2 12615953

[51] Korting HC Schöllmann C Stauss-Grabo M Schäfer-Korting M Antimicrobial peptides and skin: a paradigm of translational medicine. Skin Pharmacol Physiol (2012) 25(6):323–34 10.1159/000341990 22964878

[52] Dawson MJ Scott RW New horizons for host defense peptides and lantibiotics. Curr Opin Pharmacol (2012) 12(5):545–50 10.1016/j.coph.2012.06.006 22776251 PMC3466353

[53] Thomsen IP Dumont AL James DB Yoong P Saville BR Soper N Children with invasive *Staphylococcus aureus* disease exhibit a potently neutralizing antibody response to the cytotoxin LukAB. Infect Immun (2014) 82(3):1234–42 10.1128/IAI.01558-13 24379282 PMC3957992

[54] Barnett LG Simkins HM Barnett BE Korn LL Johnson AL Wherry EJ B cell antigen presentation in the initiation of follicular helper T cell and germinal center differentiation. J Immunol (2014) 192(8):3607–17 10.4049/jimmunol.1301284 24646739 PMC4380085

[55] Harvey BP Raycroft MT Quan TE Rudenga BJ Roman RM Craft J Transfer of antigen from human B cells to dendritic cells. Mol Immunol (2014) 58(1):56–65 10.1016/j.molimm.2013.10.013 24309484 PMC4234097

[56] Rodriguez-Pinto D Saravia NG McMahon-Pratt D CD4 T cell activation by B cells in human Leishmania (Viannia) infection. BMC Infect Dis (2014) 14:108 10.1186/1471-2334-14-108 24568275 PMC3937821

[57] Pancari G Fan H Smith S Joshi A Haimbach R Clark D Characterization of the mechanism of protection mediated by CS-D7, a monoclonal antibody to *Staphylococcus aureus* iron regulated surface determinant B (IsdB). Front Cell Infect Microbiol (2012) 2:36 10.3389/fcimb.2012.00036 22919628 PMC3417506

[58] Lambris JD Ricklin D Geisbrecht BV Complement evasion by human pathogens. Nat Rev Microbiol (2008) 6(2):132–42 10.1038/nrmicro1824 18197169 PMC2814840

[59] Gasque P Complement: a unique innate immune sensor for danger signals. Mol Immunol (2004) 41(11):1089–98 10.1016/j.molimm.2004.06.011 15476920

[60] Quilty S Kwok G Hajkowicz K Currie B High incidence of methicillin-resistant *Staphylococcus aureus* sepsis and death in patients with febrile neutropenia at Royal Darwin Hospital. Intern Med J (2009) 39(8):557–9 10.1111/j.1445-5994.2009.02003.x 19732205

[61] Swati M Gita N Sujata B Farah J Preeti M Microbial etiology of febrile neutropenia. Indian J Hematol Blood Transfus (2010) 26(2):49–55 10.1007/s12288-010-0029-z 21629636 PMC3002059

[62] Morris PG Hassan T McNamara M Hassan A Wiig R Grogan L Emergence of MRSA in positive blood cultures from patients with febrile neutropenia – a cause for concern. Support Care Cancer (2008) 16(9):1085–8 10.1007/s00520-007-0398-5 18274787

[63] Shaw BE Boswell T Byrne JL Yates C Russell NH Clinical impact of MRSA in a stem cell transplant unit: analysis before, during and after an MRSA outbreak. Bone Marrow Transplant (2007) 39(10):623–9 10.1038/sj.bmt.1705654 17384657

[64] Roos D de Boer M Molecular diagnosis of chronic granulomatous disease. Clin Exp Immunol (2014) 175(2):139–49 10.1111/cei.12202 24016250 PMC3892405

[65] Song E Jaishankar GB Saleh H Jithpratuck W Sahni R Krishnaswamy G Chronic granulomatous disease: a review of the infectious and inflammatory complications. Clin Mol Allergy (2011) 9(1):10 10.1186/1476-7961-9-10 21624140 PMC3128843

[66] Holland SM DeLeo FR Elloumi HZ Hsu AP Uzel G Brodsky N STAT3 mutations in the hyper-IgE syndrome. N Engl J Med (2007) 357(16):1608–19 10.1056/NEJMoa073687 17881745

[67] White CJ Gallin JI Phagocyte defects. Clin Immunol Immunopathol (1986) 40(1):50–61 10.1016/0090-1229(86)90068-1 2941193

[68] Badolato R Defects of leukocyte migration in primary immunodeficiencies. Eur J Immunol (2013) 43(6):1436–40 10.1002/eji.201243155 23630104

[69] Schmidt S Moser M Sperandio M The molecular basis of leukocyte recruitment and its deficiencies. Mol Immunol (2013) 55(1):49–58 10.1016/j.molimm.2012.11.006 23253941

[70] Greenlee-Wacker MC Rigby KM Kobayashi SD Porter AR DeLeo FR Nauseef WM Phagocytosis of *Staphylococcus aureus* by human neutrophils prevents macrophage efferocytosis and induces programmed necrosis. J Immunol (2014) 192(10):4709–17 10.4049/jimmunol.1302692 24729616 PMC4011196

[71] Schindler D Gutierrez MG Beineke A Rauter Y Rohde M Foster S Dendritic cells are central coordinators of the host immune response to *Staphylococcus aureus* bloodstream infection. Am J Pathol (2012) 181(4):1327–37 10.1016/j.ajpath.2012.06.039 22885107

[72] Sun K Metzger DW Influenza infection suppresses NADPH oxidase-dependent phagocytic bacterial clearance and enhances susceptibility to secondary methicillin-resistant *Staphylococcus aureus* infection. J Immunol (2014) 192(7):3301–7 10.4049/jimmunol.1303049 24563256 PMC3965630

[73] Doisne JM Tan TC Colucci F Guardian of the genome turns on genes that alert natural killer cells. Cell Cycle (2011) 10(22):3822–3 10.4161/cc.10.22.18196 22142860

[74] Wallemacq H Bedoret D Pujol J Desmet C Drion PV Farnir F CD40 triggering induces strong cytotoxic T lymphocyte responses to heat-killed *Staphylococcus aureus* immunization in mice: a new vaccine strategy for staphylococcal mastitis. Vaccine (2012) 30(12):2116–24 10.1016/j.vaccine.2012.01.039 22285272

[75] van Belkum A Melles DC Nouwen J van Leeuwen WB van Wamel W Vos MC Co-evolutionary aspects of human colonisation and infection by *Staphylococcus aureus*. Infect Genet Evol (2009) 9(1):32–47 10.1016/j.meegid.2008.09.012 19000784

[76] van Gils EJ Hak E Veenhoven RH Rodenburg GD Bogaert D Bruin JP Effect of seven-valent pneumococcal conjugate vaccine on *Staphylococcus aureus* colonisation in a randomised controlled trial. PLoS One (2011) 6(6):e20229 10.1371/journal.pone.0020229 21695210 PMC3112202

[77] Mina MJ McCullers JA Klugman KP Live attenuated influenza vaccine enhances colonization of *Streptococcus pneumoniae* and *Staphylococcus aureus* in mice. MBio (2014) 5(1):e1040–1013 10.1128/mBio.01040-13 24549845 PMC3944816

[78] Eloe-Fadrosh EA McArthur MA Seekatz AM Drabek EF Rasko DA Sztein MB Impact of oral typhoid vaccination on the human gut microbiota and correlations with *S. typhi*-specific immunological responses. PLoS One (2013) 8(4):e62026 10.1371/journal.pone.0062026 23637957 PMC3634757

[79] Ferreira RB Antunes LC Finlay BB Should the human microbiome be considered when developing vaccines? PLoS Pathog (2010) 6(11):e1001190 10.1371/journal.ppat.1001190 21124987 PMC2987818

[80] Baquir B Lin L Ibrahim AS Fu Y Avanesian V Tu A Immunological reactivity of blood from healthy humans to the rAls3p-N vaccine protein. J Infect Dis (2010) 201(3):473–7 10.1086/649901 20039802 PMC2812804

[81] Schmidt CS White CJ Ibrahim AS Filler SG Fu Y Yeaman MR NDV-3, a recombinant alum-adjuvanted vaccine for *Candida* and *Staphylococcus aureus*, is safe and immunogenic in healthy adults. Vaccine (2012) 30(52):7594–600 10.1016/j.vaccine.2012.10.038 23099329 PMC3513491

[82] Harro CD Betts RF Hartzel JS Onorato MT Lipka J Smugar SS The immunogenicity and safety of different formulations of a novel *Staphylococcus aureus* vaccine (V710): results of two phase I studies. Vaccine (2012) 30(9):1729–36 10.1016/j.vaccine.2011.12.045 22192849

[83] Ibarz-Pavón AB Maclennan J Andrews NJ Gray SJ Urwin R Clarke SC Changes in serogroup and genotype prevalence among carried meningococci in the United Kingdom during vaccine implementation. J Infect Dis (2011) 204(7):1046–53 10.1093/infdis/jir466 21881120 PMC3164428

[84] Feikin DR Kagucia EW Loo JD Link-Gelles R Puhan MA Cherian T Serotype-specific changes in invasive pneumococcal disease after pneumococcal conjugate vaccine introduction: a pooled analysis of multiple surveillance sites. PLoS Med (2013) 10(9):e1001517 10.1371/journal.pmed.1001517 24086113 PMC3782411

[85] Shirtliff ME Peters BM Jabra-Rizk MA Cross-kingdom interactions: *Candida albicans* and bacteria. FEMS Microbiol Lett (2009) 299(1):1–8 10.1111/j.1574-6968.2009.01668.x 19552706 PMC4406406

[86] Chotirmall SH O’Donoghue E Bennett K Gunaratnam C O’Neill SJ McElvaney NG Sputum *Candida albicans* presages FEV(1) decline and hospital-treated exacerbations in cystic fibrosis. Chest (2010) 138(5):1186–95 10.1378/chest.09-2996 20472859

[87] Baldan R Cigana C Testa F Bianconi I De Simone M Pellin D Adaptation of *Pseudomonas aeruginosa* in cystic fibrosis airways influences virulence of *Staphylococcus aureus* in vitro and murine models of co-infection. PLoS One (2014) 9(3):e89614 10.1371/journal.pone.0089614 24603807 PMC3945726

[88] Shinefield H Black S Fattom A Horwith G Rasgon S Ordonez J Use of a *Staphylococcus aureus* conjugate vaccine in patients receiving hemodialysis. N Engl J Med (2002) 346(7):491–6 10.1056/NEJMoa011297 11844850

[89] Fowler VG Allen KB Moreira ED Moustafa M Isgro F Boucher HW Effect of an investigational vaccine for preventing *Staphylococcus aureus* infections after cardiothoracic surgery: a randomized trial. JAMA (2013) 309(13):1368–78 10.1001/jama.2013.3010 23549582

[90] Boles JW Pitt ML LeClaire RD Gibbs PH Torres E Dyas B Generation of protective immunity by inactivated recombinant staphylococcal enterotoxin B vaccine in nonhuman primates and identification of correlates of immunity. Clin Immunol (2003) 108(1):51–9 10.1016/S1521-6616(03)00066-4 12865071

[91] Stiles BG Garza AR Ulrich RG Boles JW Mucosal vaccination with recombinantly attenuated staphylococcal enterotoxin B and protection in a murine model. Infect Immun (2001) 69(4):2031–6 10.1128/IAI.69.4.2031-2036.2001 11254555 PMC98127

[92] Anderson AS Miller AA Donald RG Scully IL Nanra JS Cooper D Development of a multicomponent *Staphylococcus aureus* vaccine designed to counter multiple bacterial virulence factors. Hum Vaccin Immunother (2012) 8(11):1585–94 10.4161/hv.21872 22922765 PMC3601133

[93] Fattom AI Horwith G Fuller S Propst M Naso R Development of StaphVAX, a polysaccharide conjugate vaccine against *S. aureus* infection: from the lab bench to phase III clinical trials. Vaccine (2004) 22(7):880–7 10.1016/j.vaccine.2003.11.034 15040941

[94] O’Brien CN Guidry AJ Fattom A Shepherd S Douglass LW Westhoff DC Production of antibodies to *Staphylococcus aureus* serotypes 5, 8, and 336 using poly(DL-lactide-co-glycolide) microspheres. J Dairy Sci (2000) 83(8):1758–66 10.3168/jds.S0022-0302(00)75046-6 10984152

[95] Zecconi A Scali F *Staphylococcus aureus* virulence factors in evasion from innate immune defenses in human and animal diseases. Immunol Lett (2013) 150(1–2):12–22 10.1016/j.imlet.2013.01.004 23376548

[96] Kraus D Peschel A *Staphylococcus aureus* evasion of innate antimicrobial defense. Future Microbiol (2008) 3(4):437–51 10.2217/17460913.3.4.437 18651815

[97] Rigby KM DeLeo FR Neutrophils in innate host defense against *Staphylococcus aureus* infections. Semin Immunopathol (2012) 34(2):237–59 10.1007/s00281-011-0295-3 22080185 PMC3271231

[98] Skurnik D Kropec A Roux D Theilacker C Huebner J Pier GB Natural antibodies in normal human serum inhibit *Staphylococcus aureus* capsular polysaccharide vaccine efficacy. Clin Infect Dis (2012) 55(9):1188–97 10.1093/cid/cis624 22806596 PMC3529611

[99] Ehrhardt GR Hijikata A Kitamura H Ohara O Wang JY Cooper MD Discriminating gene expression profiles of memory B cell subpopulations. J Exp Med (2008) 205(8):1807–17 10.1084/jem.20072682 18625746 PMC2525601

[100] Kobrynski LJ Tanimune L Kilpatrick L Campbell DE Douglas SD Production of T-helper cell subsets and cytokines by lymphocytes from patients with chronic mucocutaneous candidiasis. Clin Diagn Lab Immunol (1996) 3(6):740–5 8914768 10.1128/cdli.3.6.740-745.1996 PMC170440

[101] Turner DL Gordon CL Farber DL Tissue-resident T cells, in situ immunity and transplantation. Immunol Rev (2014) 258(1):150–66 10.1111/imr.12149 24517432

[102] Fowler VG Jr Proctor RA Where does a *Staphylococcus aureus* vaccine stand? Clin Microbiol Infect (2014) 20(Suppl 5):66–75 10.1111/1469-0691.12570 24476315 PMC4067250

[103] Schreiner J Kretschmer D Klenk J Otto M Bühring HJ Stevanovic S *Staphylococcus aureus* phenol-soluble modulin peptides modulate dendritic cell functions and increase in vitro priming of regulatory T cells. J Immunol (2013) 190(7):3417–26 10.4049/jimmunol.1202563 23460735 PMC3608756

[104] Ardura MI Banchereau R Mejias A Di Pucchio T Glaser C Allantaz F Enhanced monocyte response and decreased central memory T cells in children with invasive *Staphylococcus aureus* infections. PLoS One (2009) 4(5):e5446 10.1371/journal.pone.0005446 19424507 PMC2676512

[105] Rabe H Nordström I Andersson K Lundell AC Rudin A *Staphylococcus aureus* convert neonatal conventional CD4(+) T cells into FOXP3(+) CD25(+) CD127(low) T cells via the PD-1/PD-L1 axis. Immunology (2014) 141(3):467–81 10.1111/imm.12209 24708420 PMC3930383

[106] Chen Z Han Y Gu Y Liu Y Jiang Z Zhang M CD11c(high)CD8+ regulatory T cell feedback inhibits CD4 T cell immune response via Fas ligand-Fas pathway. J Immunol (2013) 190(12):6145–54 10.4049/jimmunol.1300060 23677464

[107] Farkas T Thornton SA Wilton N Zhong W Altaye M Jiang X Homologous versus heterologous immune responses to Norwalk-like viruses among crew members after acute gastroenteritis outbreaks on 2 US Navy vessels. J Infect Dis (2003) 187(2):187–93 10.1086/367809 12552443

[108] Díaz I Gimeno M Darwich L Navarro N Kuzemtseva L López S Characterization of homologous and heterologous adaptive immune responses in porcine reproductive and respiratory syndrome virus infection. Vet Res (2012) 43:30 10.1186/1297-9716-43-30 22515169 PMC3403850

[109] Nabel GJ Fauci AS Induction of unnatural immunity: prospects for a broadly protective universal influenza vaccine. Nat Med (2010) 16(12):1389–91 10.1038/nm1210-1389 21135852

[110] Casadevall A Pirofski LA Antibody-mediated regulation of cellular immunity and the inflammatory response. Trends Immunol (2003) 24(9):474–8 10.1016/S1471-4906(03)00228-X 12967670

[111] DeJonge M Burchfield D Bloom B Duenas M Walker W Polak M Clinical trial of safety and efficacy of INH-A21 for the prevention of nosocomial staphylococcal bloodstream infection in premature infants. J Pediatr (2007) 151(3):260–5, 265.e1 10.1016/j.jpeds.2007.04.060 17719934

[112] Burnside K Lembo A Harrell MI Klein JA Lopez-Guisa J Siegesmund AM Vaccination with a UV-irradiated genetically attenuated mutant of *Staphylococcus aureus* provides protection against subsequent systemic infection. J Infect Dis (2012) 206(11):1734–44 10.1093/infdis/jis579 22966130 PMC3488195

[113] Hua L Hilliard JJ Shi Y Tkaczyk C Cheng LI Yu X Assessment of an anti-alpha-toxin monoclonal antibody for prevention and treatment of *Staphylococcus aureus*-induced pneumonia. Antimicrob Agents Chemother (2014) 58(2):1108–17 10.1128/AAC.02190-13 24295977 PMC3910899

[114] Ragle BE Bubeck Wardenburg J Anti-alpha-hemolysin monoclonal antibodies mediate protection against *Staphylococcus aureus* pneumonia. Infect Immun (2009) 77(7):2712–8 10.1128/IAI.00115-09 19380475 PMC2708543

[115] Mocca CP Brady RA Burns DL Role of antibodies in protection elicited by active vaccination with genetically inactivated alpha hemolysin in a mouse model of *Staphylococcus aureus* skin and soft tissue infections. Clin Vaccine Immunol (2014) 21(5):622–7 10.1128/CVI.00051-14 24574539 PMC4018873

[116] Yoong P Pier GB Antibody-mediated enhancement of community-acquired methicillin-resistant *Staphylococcus aureus* infection. Proc Natl Acad Sci U S A (2010) 107(5):2241–6 10.1073/pnas.0910344107 20133867 PMC2836673

[117] Weems JJ Jr Steinberg JP Filler S Baddley JW Corey GR Sampathkumar P Phase II, randomized, double-blind, multicenter study comparing the safety and pharmacokinetics of tefibazumab to placebo for treatment of *Staphylococcus aureus* bacteremia. Antimicrob Agents Chemother (2006) 50(8):2751–5 10.1128/AAC.00096-06 16870768 PMC1538656

[118] Proctor RA Challenges for a universal *Staphylococcus aureus* vaccine. Clin Infect Dis (2012) 54(8):1179–86 10.1093/cid/cis033 22354924

[119] Seok J Warren HS Cuenca AG Mindrinos MN Baker HV Xu W Genomic responses in mouse models poorly mimic human inflammatory diseases. Proc Natl Acad Sci U S A (2013) 110(9):3507–12 10.1073/pnas.1222878110 23401516 PMC3587220

[120] Kim HK Missiakas D Schneewind O Mouse models for infectious diseases caused by *Staphylococcus aureus*. J Immunol Methods (2014) 410:88–99 10.1016/j.jim.2014.04.007 24769066 PMC6211302

[121] Sung JM Lloyd DH Lindsay JA *Staphylococcus aureus* host specificity: comparative genomics of human versus animal isolates by multi-strain microarray. Microbiology (2008) 154(Pt 7):1949–59 10.1099/mic.0.2007/015289-0 18599823

[122] Jansen KU Girgenti DQ Scully IL Anderson AS Vaccine review: “*Staphylococcus aureus* vaccines: problems and prospects”. Vaccine (2013) 31(25):2723–30 10.1016/j.vaccine.2013.04.002 23624095

[123] Ahn SH Tsalik EL Cyr DD Zhang Y van Velkinburgh JC Langley RJ Gene expression-based classifiers identify *Staphylococcus aureus* infection in mice and humans. PLoS One (2013) 8(1):e48979 10.1371/journal.pone.0048979 23326304 PMC3541361

[124] Date SV Modrusan Z Lawrence M Morisaki JH Toy K Shah IM Global gene expression of methicillin-resistant *Staphylococcus aureus* USA300 during human and mouse infection. J Infect Dis (2014) 209(10):1542–50 10.1093/infdis/jit668 24286981

[125] Casadevall A Pirofski LA Exploiting the redundancy in the immune system: vaccines can mediate protection by eliciting “unnatural” immunity. J Exp Med (2003) 197(11):1401–4 10.1084/jem.20030637 12782708 PMC2193913

[126] Jenner E On Vaccination Against Smallpox: An Inquiry into the Causes and Effects of the Variole Vaccine, or Cow Pox. London: Sampson Low (1798).

[127] Murphy SG Tetanus toxin and antigenic derivatives. II. Effect of protein and formaldehyde concentration on toxoid formation. J Bacteriol (1967) 94(3):586–9 6035261 10.1128/jb.94.3.586-589.1967 PMC251927

[128] Novitsky V Smith UR Gilbert P McLane MF Chigwedere P Williamson C Human immunodeficiency virus type 1 subtype C molecular phylogeny: consensus sequence for an AIDS vaccine design? J Virol (2002) 76(11):5435–51 10.1128/JVI.76.11.5435-5451.2002 11991972 PMC137027

[129] Koita OA Dabitao D Mahamadou I Tall M Dao S Tounkara A Confirmation of immunogenic consensus sequence HIV-1 T-cell epitopes in Bamako, Mali and Providence, Rhode Island. Hum Vaccin (2006) 2(3):119–28 10.4161/hv.2869 17012903

[130] Giles BM Ross TM A computationally optimized broadly reactive antigen (COBRA) based H5N1 VLP vaccine elicits broadly reactive antibodies in mice and ferrets. Vaccine (2011) 29(16):3043–54 10.1016/j.vaccine.2011.01.100 21320540 PMC3090662

[131] Hausdorff WP Hoet B Schuerman L Do pneumococcal conjugate vaccines provide any cross-protection against serotype 19A? BMC Pediatr (2010) 10:4 10.1186/1471-2431-10-4 20122261 PMC2829470

[132] McNeely TB Staub JM Rusk CM Blum MJ Donnelly JJ Antibody responses to capsular polysaccharide backbone and O-acetate side groups of *Streptococcus pneumoniae* type 9V in humans and rhesus macaques. Infect Immun (1998) 66(8):3705–10 9673252 10.1128/iai.66.8.3705-3710.1998 PMC108405

[133] Jakobsen H Sigurdsson VD Sigurdardottir S Schulz D Jonsdottir I Pneumococcal serotype 19F conjugate vaccine induces cross-protective immunity to serotype 19A in a murine pneumococcal pneumonia model. Infect Immun (2003) 71(5):2956–9 10.1128/IAI.71.5.2956-2959.2003 12704178 PMC153277

[134] Richter SS Heilmann KP Dohrn CL Riahi F Diekema DJ Doern GV Pneumococcal serotypes before and after introduction of conjugate vaccines, United States, 1999-2011(1.). Emerg Infect Dis (2013) 19(7):1074–83 10.3201/eid1907.121830 23763847 PMC3713983

[135] Hulten KG Kaplan SL Lamberth LB Barson WJ Romero JR Lin PL Changes in *Streptococcus pneumoniae* serotype 19A invasive infections in children from 1993 to 2011. J Clin Microbiol (2013) 51(4):1294–7 10.1128/JCM.00058-13 23390277 PMC3666798

[136] Dudas RA Karron RA Respiratory syncytial virus vaccines. Clin Microbiol Rev (1998) 11(3):430–9 9665976 10.1128/cmr.11.3.430 PMC88889

[137] Fu Y Ibrahim AS Sheppard DC Chen YC French SW Cutler JE *Candida albicans* Als1p: an adhesin that is a downstream effector of the EFG1 filamentation pathway. Mol Microbiol (2002) 44:61–72 10.1046/j.1365-2958.2002.02873.x 11967069

[138] Phan QT Myers CL Fu Y Sheppard DC Yeaman MR Welch WH Als3 is a *Candida albicans* invasin that binds to cadherins and induces endocytosis by host cells. PLoS Biol (2007) 5(3):e64 10.1371/journal.pbio.0050064 17311474 PMC1802757

[139] Sheppard DC Yeaman MR Welch WH Phan QT Fu Y Ibrahim AS Functional and structural diversity in the Als protein family of *Candida albicans*. J Biol Chem (2004) 279:30480–9 10.1074/jbc.M401929200 15128742

[140] Lin L Ibrahim AS Xu X Farber JM Avanesian V Baquir B Th1-Th17 cells mediate protective adaptive immunity against *Staphylococcus aureus* and *Candida albicans* infection in mice. PLoS Pathog (2009) 5(12):e1000703 10.1371/journal.ppat.1000703 20041174 PMC2792038

[141] Yeaman MR Filler SG Schmidt CS Chaili S Barr K Wang H Efficacy and immunologic mechanisms of NDV-3 vaccine in a murine model of MRSA skin/skin structure infection. 52nd ICAAC. San Francisco, CA (2012). Abstract No. G-868.

[142] Festa M Brun P Piccinini R Castagliuolo I Basso B Zecconi A *Staphylococcus aureus* Efb protein expression in *Nicotiana tabacum* and immune response to oral administration. Res Vet Sci (2013) 94(3):484–9 10.1016/j.rvsc.2012.10.012 23158852

[143] Camussone CM Veaute CM Pujato N Morein B Marcipar IS Calvinho LF Immune response of heifers against a *Staphylococcus aureus* CP5 whole cell and lysate vaccine formulated with ISCOM matrix adjuvant. Res Vet Sci (2014) 96(1):86–94 10.1016/j.rvsc.2013.10.004 24210331

[144] Camussone CM Veaute CM Porporatto C Morein B Marcipar IS Calvinho LF Immune response of heifers against a *Staphylococcus aureus* CP5 whole cell vaccine formulated with ISCOMATRIX adjuvant. J Dairy Res (2013) 80(1):72–80 10.1017/S0022029912000593 23171590

[145] Luo G Hennessey JP Jr Schmidt CS Fu Y Yeaman MR Filler SG Human vaccination with rAls3p-N produces antibodies that enhance phagocyte-mediated killing of *C. albicans* and *S. aureus* and mitigate Als3p functions. 18th Congress of the International Society for Human and Animal Mycology. Berlin (2012).

[146] Klotz SA Chasin BS Powell B Gaur NK Lipke PN Polymicrobial bloodstream infections involving *Candida* species: analysis of patients and review of the literature. Diagn Microbiol Infect Dis (2007) 59(4):401–6 10.1016/j.diagmicrobio.2007.07.001 17888612

[147] Spellberg BJ Ibrahim AS Avanesian V Fu Y Myers C Phan QT Efficacy of the anti-*Candida* rAls3p-N or rAls1p-N vaccines against disseminated and mucosal candidiasis. J Infect Dis (2006) 194(2):256–60 10.1086/504691 16779733

[148] Ibrahim AS Luo G Gebremariam T Lee H Schmidt CS Hennessey JP Jr NDV-3 protects mice from vulvovaginal candidiasis through T- and B-cell immune response. Vaccine (2013) 31(47):5549–56 10.1016/j.vaccine.2013.09.016 24063977 PMC3866209

[149] Bär E Gladiator A Bastidas S Roschitzki B Acha-Orbea H Oxenius A A novel Th cell epitope of *Candida albicans* mediates protection from fungal infection. J Immunol (2012) 188(11):5636–43 10.4049/jimmunol.1200594 22529294

[150] Pentier JM Sewell AK Miles JJ Advances in T-cell epitope engineering. Front Immunol (2013) 4:133 10.3389/fimmu.2013.00133 23761792 PMC3672776

[151] McNeil SA Halperin SA Langley JM Smith B Warren A Sharratt GP Safety and immunogenicity of 26-valent group a *Streptococcus* vaccine in healthy adult volunteers. Clin Infect Dis (2005) 41(8):1114–22 10.1086/444458 16163629

[152] Zeng W Horrocks KJ Robevska G Wong CY Azzopardi K Tauschek M A modular approach to assembly of totally synthetic self-adjuvanting lipopeptide-based vaccines allows conformational epitope building. J Biol Chem (2011) 286(15):12944–51 10.1074/jbc.M111.227744 21321114 PMC3075641

[153] Sahdev P Ochyl LJ Moon JJ Biomaterials for nanoparticle vaccine delivery systems. Pharm Res (2014). 10.1007/s11095-014-1419-y 24848341 PMC4198431

[154] Gregory AE Titball R Williamson D Vaccine delivery using nanoparticles. Front Cell Infect Microbiol (2013) 3:13 10.3389/fcimb.2013.00013 23532930 PMC3607064

[155] Lambrecht BN Hammad H Biology of lung dendritic cells at the origin of asthma. Immunity (2009) 31(3):412–24 10.1016/j.immuni.2009.08.008 19766084

[156] Vander Lugt B Khan AA Hackney JA Agrawal S Lesch J Zhou M Transcriptional programming of dendritic cells for enhanced MHC class II antigen presentation. Nat Immunol (2014) 15(2):161–7 10.1038/ni.2795 24362890

[157] Cruz LJ Rueda F Simón L Cordobilla B Albericio F Domingo JC Liposomes containing NYESO1/tetanus toxoid and adjuvant peptides targeted to human dendritic cells via the Fc receptor for cancer vaccines. Nanomedicine (Lond) (2014) 9(4):435–49 10.2217/NNM.13.66 24910875

[158] Kaslow DC Shiver JW Clostridium difficile and methicillin-resistant *Staphylococcus aureus*: emerging concepts in vaccine development. Annu Rev Med (2011) 62:201–15 10.1146/annurev-med-051109-101544 20707676

[159] Proctor RA Is there a future for a *Staphylococcus aureus* vaccine? Vaccine (2012) 30(19):2921–7 10.1016/j.vaccine.2011.11.006 22115633

